# Investigation of membrane fouling mechanism of intracellular organic matter during ultrafiltration

**DOI:** 10.1038/s41598-020-79272-4

**Published:** 2021-01-13

**Authors:** Weiwei Huang, Yuanhong Zhu, Bingzhi Dong, Weiwei Lv, Quan Yuan, Wenzong Zhou, Weiguang Lv

**Affiliations:** 1 Eco-Environmental Protection Research Institute, Shanghai Academy of Agricultural Sciences, Shanghai, 201403 China; 2 School of Environmental Science and Engineering, Tongji University, Shanghai, 200092 China; 3 Shanghai Qingpu Modern Agriculture Park, Shanghai, 201403 China

**Keywords:** Environmental impact, Civil engineering

## Abstract

This study investigated the ultrafiltration (UF) membrane fouling mechanism of intracellular organic matter (IOM) from *Chlorella vulgaris* (CV) and *Microcystis aeruginosa* (MA). Both CV- and MA-IOM caused severe membrane fouling during UF; however, there were significant differences in the membrane fouling by these two materials. Neutral hydrophilic (N-HPI) compounds were the organics that caused the most severe membrane fouling during CV-IOM filtration, whereas the MA-IOM membrane fouling was induced by mainly hydrophobic (HPO) organics. From an analysis based on Derjaguin–Landau–Verwey–Overbeek theory, it was found that the interaction energy between the membrane and foulants in the later stage of filtration was the major factor determining the efficiency of filtration for both CV-IOM and MA-IOM. The TPI organics in CV-IOM fouled the membrane to a more severe degree during the initial filtration flux; however, when the membrane surface was covered with CV-IOM foulants, the N-HPI fraction of CV-IOM caused the most severe membrane fouling because its attractive energy with the membrane was the highest. For MA-IOM, regardless of the initial filtration flux or the late stage of filtration, the HPO organics fouled the membrane to the greatest extent. An analysis of modified filtration models revealed that cake layer formation played a more important role than other fouling mechanisms during the filtration of CV-IOM and MA-IOM. This study provides a significant understanding of the membrane fouling mechanism of IOM and is beneficial for developing some strategies for membrane fouling control when treating MA and CV algae-laden waters.

## Introduction

Water is an indispensable resource for human life and ecosystems, however, the nutrients released via the discharge of sewage and leaching of fertilizer into lakes and reservoirs has caused extensive eutrophication^1^. Every year, millions of deaths in poorer countries result from a lack of clean water, and in most affluent countries, the demand for such water is continuously increasing. Microalgae, which are generally detected as blooms, have become a worldwide problem in water treatment. Because the frequent outbreak of algal blooms can produce serious water quality problems, such as odors, toxins, and disinfection byproducts, and increase public concerns about water safety, many water treatment chemicals have been applied to treat algae-rich water^2,3^. However, owing to the properties of the algae and the intracellular organic matter (IOM) and toxins that might be released, such chemicals may result in human health hazards^4^.

Ultrafiltration (UF) can be used to treat algae-rich water and has drawn increasing attention for algal biomass harvesting. During UF, algal cells are rejected from the filter, but the deposition of algal cells and released algogenic organic matter (AOM) on the membrane surface inevitably foul the membrane, limiting the application of UF for water treatment^5^. Many studies have been conducted on membrane fouling. Zhang et al. studied the membrane fouling in aerobic granular sludge (AGS)-membrane bioreactor (MBR) and found that AGS size had great relations with membrane fouling, and there existed a maximum membrane fouling at the critical AGS size, besides, it was indicated that transparent exopolymer particles (TEP) can affect membrane fouling^6–8^. AOM is also considered as one of the most important foulants^9,10^. Generally, AOM can be divided into extracellular organic matter (EOM) and IOM. IOM is released into natural waters following algal cell death in aquatic ecosystems and/or during the dead phase, peroxidation, hydraulic shearing, and other external stress-induced processes.

Previous research suggested that IOM, which is composed of high-molecular-weight proteins and polysaccharides, could promote the formation of disinfection byproducts (DBPs) and increase the color and the taste and odor levels^11^. Compared with the widely researched EOM^12,13^, IOM is less understood in terms of its effect on UF. Li et al. studied the influence of EOM and IOM from *Microcystis aeruginosa* (MA) on UF and found that IOM is more likely to form a compacted cake layer than to penetrate the membrane pores^14^. In addition, it was indicated that IOM could cause severe declines in the filtration flux^5^. Nevertheless, as the intracellular organic characteristics were complicated, which might be influenced by algal species, algal growth time, as well as other factors that might have significant effects on membrane fouling. A better understanding of the effects of IOM on membrane fouling is needed to improve the strategies for membrane fouling control. However, as noted above, the membrane fouling mechanism of IOM has not yet been fully explored.

Conventional natural organics matter (NOM) fouling characterization techniques have included protein or polysaccharide determination, molecular weight (MW) distribution, fluorescence excitation emission matrices (EEM), attenuated total reflection-Fourier infrared spectroscopy (ATR-FTIR), and scanning electron microscopy (SEM), despite these methods are available for organic characteristics analysis and fouling layers determination on membrane surfaces, they are generally limited to qualitative assessment^15–17^. The membrane fouling in fact can be considered an interaction between membrane materials and foulants under certain filtration modes^18^. From a microscopic perspective, these physicochemical interactions can be considered to arise from Lifshitz-van der Waals (LW) forces, electrostatic (EL) interaction energy, and acid–base (AB) interaction energy. Park et al. suggested that the extended Derjaguin–Landau–Verwey–Overbeek (XDLVO) theory was a powerful and rigorous method to further understand membrane fouling, which could quantitatively evaluate the interactions between the membrane and foulants^19^. Chen et al. found that the XDLVO theory could provide considerable insights into the role of interactions of soluble microbial products (SMP) on different membrane surfaces^20^. The alleviation of UF fouling by preoxidation on aged membrane surfaces was also studied using XDLOV theory^21,22^. However, there have been limited systematic investigations of the membrane fouling effects of IOM using the application of XDLVO theory.

This study investigated the fouling mechanisms of IOM during UF. Two types of IOM solutions were adopted considering that there are various dominant algal species in natural waters. The XDLVO theory was utilized to elucidate the effects of various fouling components. The aim of this work is to provide valuable information for ways to reduce UF fouling and guidance for membrane process design in algae-containing water treatment.

## Materials and methods

### Algae cultivation and EOM and IOM extraction

*Chlorella vulgaris* (CV) and MA were obtained from the Institute of Aquatic Biology, Chinese Academy of Sciences. The cultivation conditions consisted of 12 h of fluorescent light:12 h of darkness at 25 °C with approximately an irradiance of 90 μmol/m^2^·s in BG-11 medium. Both MA and CV were used in this experiment because there are various predominant types of blue-green algae in rivers and lakes, and the IOM characteristics might differ among various algae species.

IOM was extracted by centrifuging algal cell suspension in the stationary phase at 10,000 r/min for 15 min. The algal precipitates were washed three times, resuspended in Milli-Q water, freeze-thawed (− 80 °C in an ultralow freezer, 35 °C in a water bath) for three cycles, centrifuged at 10,000 r/min for 15 min, and then the supernatant was filtered through a 0.45 μm polyether sulfone membrane filter^14^.

### Experimental setup

**Figure 1:**
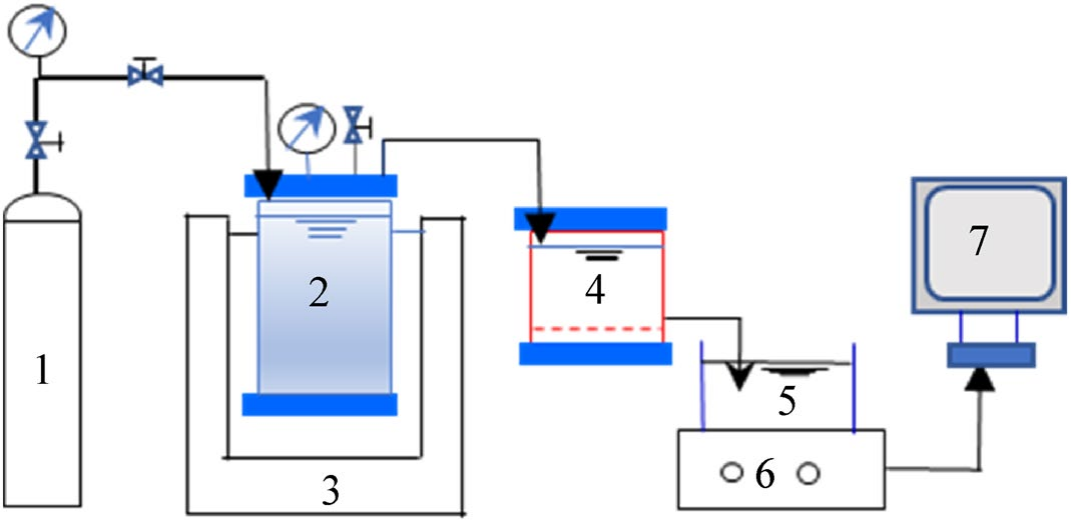
Schematic diagram of the filtration setup, 1. Nitrogen cylinder, 2. 4 L water vessel, 3. Low temperature storage tank, 4. Membrane filtration vessel, 5. Effluent, 6. Electronic balance, 7. Data record.

The UF test was performed in dead-end filtration equipment using a 400 mL stirring cell (Amicon 8400, Millipore, USA) (Fig. 1), as described in our prior research^23^. The membrane used was a flat sheet 100 kDa polyethersulfone (PES) membrane (OM100076, Pall, USA), and the surface area was 45 cm^2^. Nitrogen gas at 0.1 MPa was adopted to drive the feed solution through the membrane, and the weighting data were logged automatically on a computer every 1 min. Before UF, all clean membranes were presoaked in Milli-Q water (18.25 MΩ·cm) for at least 24 h at 4 °C and washed with 5 L of Milli-Q water until the dissolved organic carbon (DOC) of the effluent was zero. Prior to filtration, all IOM fractions were adjusted to have identical ionic strength and DOC concentrations (5 ± 0.05 mg/L) and neutralized to pH 7.0 to minimize their effects on the filtration fluxes. To ensure the reliability of the experimental data, the experiment was duplicated, and the error between the fluxes of repeated experiments was calculated.

### Analysis of membrane fouling

To analyze the membrane fouling by organic foulants on the membrane surfaces and inside the membrane pores, modified model equations from the pore blocking model, pore constriction model, and cake formation model were utilized^16^, as follows:

1 $$ {\text{Pore}}\;{\text{ blocking}}\;{\text{ model}}:\quad {\text{j}} = \exp \left( { - \propto_{b} \cdot t} \right) $$

2 $$ {\text{Pore}}\;{\text{ constriction}}\;{\text{ model}}:\quad {\text{j}} = \left( {1 + \propto_{p} t} \right)^{ - 2} $$

3 $$ {\text{Cake}}\;{\text{ formation}}\;{\text{ model}}:\quad {\text{j}} = \left( {1 + \propto_{c} t} \right)^{ - 1/2} $$

where j (dimensionless ratio) is the ratio of permeate flux (J) to the initial permeate flux (J_0_); $\propto_{b}$ (h^−1^) is the rate constant of the pore blocking model, $\propto_{p }$ (h^−1^) is the rate constant of the pore constriction model, $\propto_{c }$ (h^−1^) is the rate constant of the cake formation model, and t is the filtration time (h). The R-squared (R^2^) value was also determined, which indicated the goodness of the fit.

The rate constant can be calculated by Eqs. (4–6)^24^:

4 $$ { } - {\text{lnj}} = \propto_{b} t $$

5 $$ j^{ - 1/2} = 1 + 2 \propto_{p} t $$

6 $$ j^{ - 2} = 1 + 2 \propto_{c} t $$

In addition, the contribution factor (CF) of each rate constant can be obtained^24,25^:

7 $$ {\text{ CF}} = \frac{{ \propto_{x} }}{{\left( { \propto_{b} + \propto_{p} + \propto_{c} } \right)}} $$

where $\propto_{x}$ indicates the rate constant of each model, i.e., $\propto_{b}$, $\propto_{p}$, or $\propto_{c}$.

### XDLVO theory

#### Theoretical models

According to the XDLVO theory, the foulant-membrane interaction energy can be calculated as the sum of the LW, electrostatic double layer (EL), and Lewis acid–base interactions (AB)^26^:

8 $$ U_{fwm}^{TOT} = U_{fwm}^{LW} + U_{fwm}^{AB} + U_{fwm}^{EL} $$

The LW, AB, and EL interaction energy components between a spherical foulant and an infinite planar surface can be presented as follows^27,28^:

9 $$ U_{fwm}^{LW} = 2\pi \Delta G_{{h_{0} }}^{LW} \left( {\frac{{h_{0}^{2} a}}{h}} \right) $$

10 $$ U_{fwm}^{AB} = 2\pi a\lambda \Delta G_{{h_{0} }}^{AB} \exp \left[ {\frac{{h_{0} - h}}{\lambda }} \right] $$

11 $$ U_{fwm}^{EL} = \pi \varepsilon_{r} \varepsilon_{0} a\left[
{2\xi_{f} \xi_{m} \ln \left( {\frac{{1 + e^{ - \kappa h} }}{{1 -
e^{\kappa h} }}} \right) + (\xi_{f}^{2} + \xi_{m}^{2} )\ln (1 - e^{
- 2\kappa h} )} \right] $$

12 $$ U_{fwm}^{TOT} = U_{fwm}^{LW} + U_{fwm}^{AB} + U_{fwm}^{EL} $$

where f, w, and m indicate the foulant, water, and membrane, respectively; a denotes the foulant radius; h represents the separation distance between the membrane and foulant;$h_{0}^{{}}$ represents the minimum equilibrium separation distance (usually assigned a value of 0.158 ± 0.009 nm); ε_r_ε_0_ represents the dielectric permittivity of the suspending fluid (F/m)^22^; $\kappa$ is the inverse Debye screening length; and λ ($\cong$ 0.6 nm) is the characteristic decay length of the LW interaction. ζ_f_ and ζ_m_ are the surface potentials of the foulant and membrane, respectively.

When the separation distance between the two surfaces is close to the minimum equilibrium separation distance, the AB, LW and EL adhesion free energies per unit area can be obtained by the following^29^

13 $$  \Delta G_{{h_{0} }}^{{LW}}  = {\text{ }}\left( {\sqrt {\gamma _{w}^{{LW}} }  - {\text{ }}\sqrt {\gamma _{m}^{{LW}} } } \right)\left( {\sqrt {\gamma _{f}^{{LW}} }  - \sqrt {\gamma _{w}^{{LW}} } } \right)  $$

14 $$ \Delta G_{{h_{0} }}^{AB} = 2\left( {\sqrt {\gamma_{w}^{ + } } \left( {\sqrt {\gamma_{m}^{ - } } + \sqrt {\gamma_{f}^{ - } } - \sqrt {\gamma_{w}^{ - } } } \right) + 2\sqrt {\gamma_{w}^{ - } } \left( {\sqrt {\gamma_{m}^{ + } } + \sqrt {\gamma_{f}^{ + } } - \sqrt {\gamma_{w}^{ + } } } \right) - 2\sqrt {\gamma_{f}^{ - } \gamma_{m}^{ + } } + \sqrt {\gamma_{f}^{ + } \gamma_{m}^{ - } } } \right) $$

15 $$ \Delta G^{EL} = \frac{{\varepsilon_{0} \varepsilon_{r} \kappa }}{2}(\xi_{m}^{2} + \xi_{f}^{2} ) \times (1 - \coth (\kappa h_{0} ) + \frac{{2\xi_{m} \xi_{f} }}{{(\xi_{m}^{2} + \xi_{f}^{2} )}}{\text{csch}} (\kappa h_{0} )) $$

16 $$ \Delta G^{TOT} = \Delta G^{LW} + \Delta G^{AB} + \Delta G^{EL} $$

In addition, the surface tension parameters ($\gamma_{s}^{ - }$, $\gamma_{s}^{ + }$, and $\gamma_{s}^{LW}$) of the membrane and foulants were expressed as follows^29–32^:

17 $$ (1 + \cos \theta_{1} )\gamma_{l}^{TOT} = 2\left[\sqrt {(\gamma_{s}^{LW} \gamma_{l}^{LW} } ) + \sqrt {(\gamma_{s}^{ + } \gamma_{l}^{\_} )} + \sqrt {(\gamma_{s}^{ - } \gamma_{l}^{ + } )} \right] $$

18 $$ \gamma^{TOT} = \gamma^{LW} + \gamma^{AB} $$

19 $$ \gamma^{AB} = 2\sqrt {\gamma^{ + } \gamma^{ - } } $$

where $\Delta G_{{h_{0} }}^{LW}$, $\Delta G_{{h_{0} }}^{AB}$, $\;{\text{ and}} \;\Delta G_{{h_{0} }}^{EL}$ are the Lifshitz-van der Waals, acid–base, and electrostatic double-layer free energy components at a separation distance of h_0_; the subscript (s) represents the solid surface of the membrane or foulant; (l) represents the liquid used in each determination; θ refers to the contact angle; $\gamma^{TOT}$, $\gamma^{LW}$, $\gamma^{\_}$, and $\gamma^{ + }$ denote the total surface tension, the LW component, the electron-donor parameter, and the electron-acceptor parameter, respectively.

### Analytical methods

DOC was measured by a total organic carbon (TOC) analyzer (Shimadzu TOC-L, Japan). The contact angles of the foulants and membrane were determined by a drop shape analyzer (DSA30, KRUSS) with three different diagnostic liquids (water, glycerol, and diiodomethane) of known surface tension. Each sample was determined at least seven times.

Zeta potential and mean size were determined by dynamic light scattering (DLS) with a Malvern Zetasizer NANO ZS system (Malvern Instruments Limited, UK) equipped with a He–Ne laser (wavelength of 633 nm) with a detector angle of 173° at 25 °C. The mean hydrodynamic diameter (Z-average) was characterized by DLS cumulant analysis. Zeta potential was calculated by the Smoluchowski equation^33^. Each data point was determined at least three times.

Organic fractionation was performed using an XAD-8/XAD-4/IRA-958 (Amberlite, USA) column, which fractionated the organics into hydrophobic (HPO), transphilic (TPI), charged hydrophilic (C-HPI), and neutral hydrophilic (N-HPI) fractions according to Carroll et al.^34^. The XAD resin fractionation procedure was as follow: (1) IOM samples were acidified to pH 2 and passed through the XAD-8 and XAD-4 resins consecutively, (2) then the effluent adjusted to pH 8 was pumped into the IRA-958, (3) the organic matter of effluent not absorbed to either resins was considered as N-HPI, whereas the organics adsorbed onto XAD-8, XAD-4, and IRA-958 resins comprised of HPO, TPI, and C-HPI, respectively. The XAD-8 and XAD-4 columns were back-eluted using NaOH (0.1 mol/L), while the IRA-958 column was back-eluted with 1 mol/L NaOH and 1 mol/L NaCl. Each fractionation was completed in duplicate. The recovery rates were > 98%.

The MW distribution was measured on a high-performance size exclusion chromatograph (Waters e2695, USA) coupled with a UV/visible detector (Waters 2489, USA)-TOC analyzer (Sievers 900 Turbo, USA) system^35^. The column used was a TSKgelG3000PW XL (30 cm × 7.8 cm). A pre-column of TSK-GEL TSK guard column PW XL (6.0 mm × 4.0 cm) was adopted for the protection of TSKgelG3000PW XL column. The mobile phase was 0.05 mol/L KH_2_PO_4_, 0.03 mol/L NaOH, and 0.02 mol/L Na_2_SO_4_, creating the ionic strength of 0.1 mol/L. Prior to detection, all water samples were neutralized to pH 7.0 ± 0.1 and adjusted to ionic strength 0.1 mol/L. The standard samples were sodium polystyrene sulfonate (PSS, 3.61 kDa, 6.8 kDa, 15.45 kDa, and 31 kDa) and polyethylene glycols (PEGs, 200 Da, 1400 Da), with broad standard calibration adopted.

Surface morphology and topographical observation of fouled membranes was examined by scanning electron microscopy (SEM, XL-30ESEM, Philips, Holland) and atomic force microscopy (AFM, Veeco nanoscope 15, USA).

## Results and discussion

### Filtration behaviors of IOM fractions

**Figure 2:**
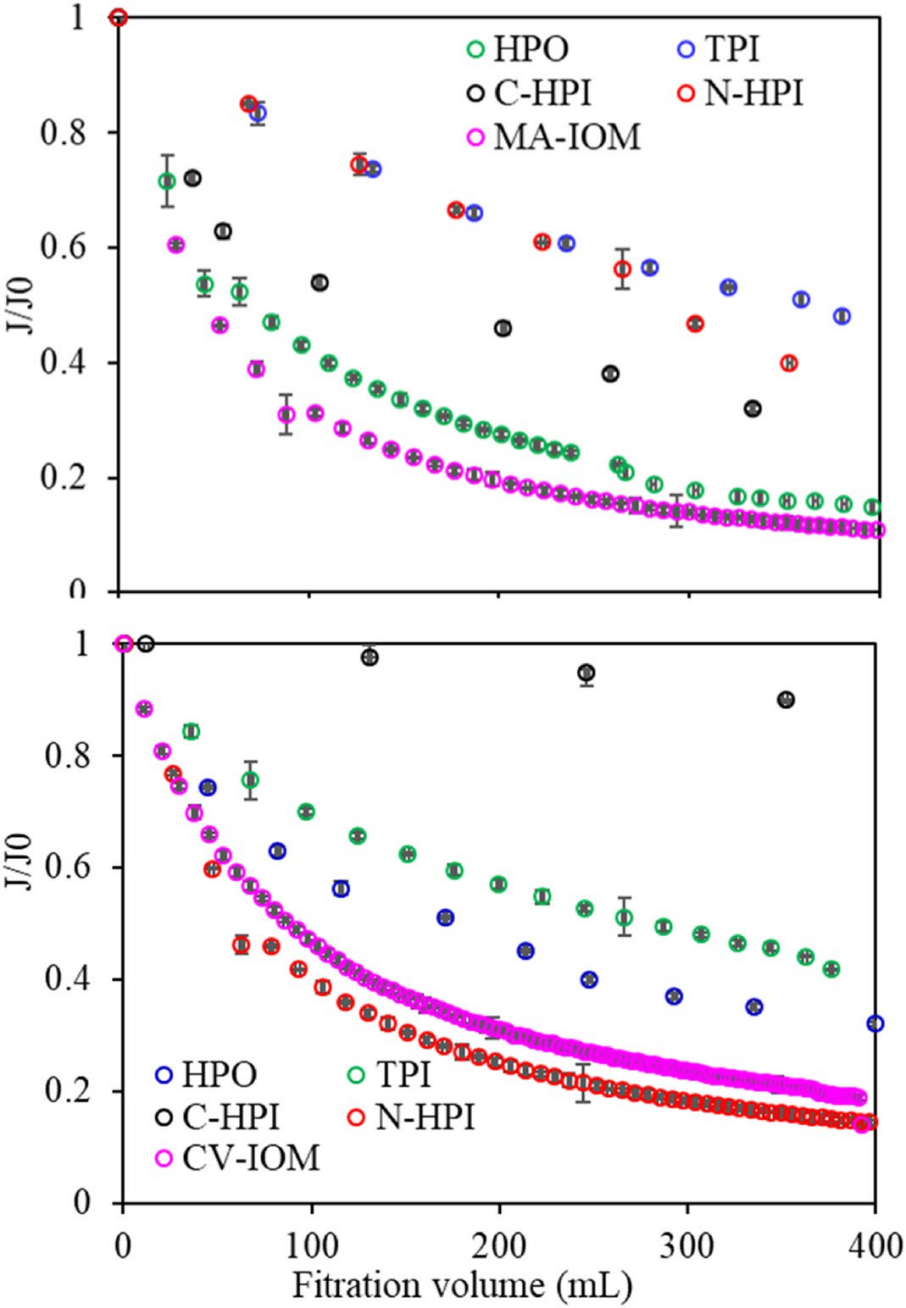
Variation of filtration flux of various IOM fractions (DOC 5 ± 0.05 mg/L, pH 7.0).

Figure 2 displays the filtration results for the CV- and MA-IOM solutions. As shown in Fig. 2, by the end of filtration, the filtration flux had decreased 18.7% with CV-IOM but only 10.8% with MA-IOM, suggesting that both CV- and MA-IOM induced severe membrane fouling during the treatment of algae-containing water, but the fouling induced by MA-IOM was more severe than that induced by CV-IOM. This result was in accordance with our previous research finding that MA-AOM resulted in more severe membrane fouling than CV-AOM did^36^, which might be due to their IOM characteristics.

The filtration fluxes with various IOM fractions were also examined. The filtration flux decline varied among the IOM fractions, decreasing by 32%, 41.7%, 86%, and 13.9% at the end of filtration for HPO, TPI, C-HPI, and N-HPI in CV-IOM, respectively. For MA-IOM, the decline was 14.8%, 48%, 32%, and 36% for HPO, TPI, C-HPI, and N-HPI, respectively, suggesting that although both MA-IOM and CV-IOM caused severe membrane fouling, the UF fouling induced was extremely different. N-HPI was the organic fraction that caused the most severe membrane fouling during CV-IOM filtration, whereas MA-IOM membrane fouling induced was mainly by HPO organics.

To further understand membrane fouling by both types of IOM, the effect of organic hydrophobicity on the flux decline mechanism was investigated by three classic filtration models^24^. As shown in Table 1, Fig. S2, and Fig. S3 (in the supporting information), the rate constants of the pore blocking mode $\propto_{b}$ (h^−1^) were 0.1329, 0.0523, 0.0261, and 0.0272 for HPO, TPI, C-HPI, and N-HPI of CV-IOM, respectively, while the pore constriction model $\propto_{p}$ values obtained were 0.0878, 0.0346, 0.0131, and 0.0269 for HPO, TPI, C-HPI, and N-HPI, respectively, and the $\propto_{c} {\text{values }}({\text{cake formation model}}$) were 0.884, 0.288, 0.0511, and 1.00226 for HPO, TPI, C-HPI, and N-HPI, respectively. For MA-IOM, $\propto_{b}$, $\propto_{p}$, and $\propto_{c}$ were 0.0573, 0.0475, and 1.0232 for HPO; 0.1023, 0.0608, and 0.4235 for TPI; 0.2328, 0.131, and 0.7686 for C-HPI; and 0.1073, 0064, and 0.4518 for N-HPI, respectively. Comparing the magnitudes of the above rate constants for both IOM fractions revealed that all the fouling rates owing to cake layer formation were the highest for the MA-IOM and CV-IOM fractions, followed by $\propto_{b}$ and $\propto_{p}$, indicating that the membrane fouling induced by both CV-IOM and MA-IOM was mostly ascribed to cake layer formation.

**Table 1:** Rate constants and R^2^ values of regression analyses of CV- and MA-IOM fraction fouling.

	Pore blocking		Pore constriction		Cake formation	
	$\propto_{b}$(h^−1^)	R^2^	$\propto_{p }$(h^−1^)	R^2^	$\propto_{c }$(h^−1^)	R^2^
CV-HPO	0.133	0.962	0.088	0.985	0.884	0.983
CV-TPI	0.052	0.940	0.035	0.943	0.288	0.963
CV-C-HPI	0.026	0.999	0.013	0.999	0.055	0.999
CV-N-HPI	0.027	0.828	0.027	0.917	1.023	0.998
MA-HPO	0.057	0.909	0.048	0.955	1.023	0.958
MA-HPI	0.102	0.966	0.061	0.981	0.424	0.999
MA-C-HPI	0.233	0.948	0.131	0.959	0.768	0.987
MA-N-HPI	0.107	0.981	0.064	0.992	0.452	0.997

**Figure 3:**
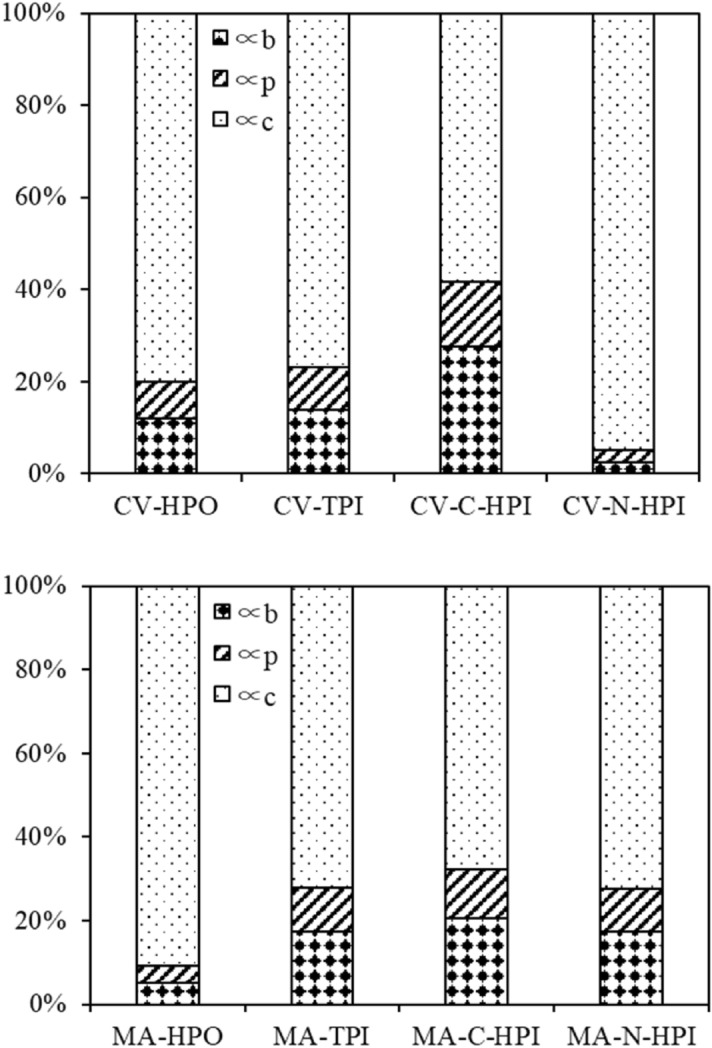
Contribution factor of each rate constant for CV-IOM and MA-IOM fractions.

The contributing factors of each rate constant for various IOM fractions were also determined (Fig. 3). It was found that the membrane fouling induced by cake formation (∝ _c_) demonstrated a comparatively higher proportion of various IOM fractions than the other mechanisms; however, comparing the contributing factors of other rate constants for the various IOM fractions shows that the proportion of ∝ _c_ differed among various fractions. The N-HPI in CV-IOM had the highest proportion of ∝ _c_, followed by HPO, TPI, and C-HPI, while for MA-IOM, the highest proportion of ∝ _c_ appeared in HPO, followed by N-HPI, C-HPI, and TPI, consistent with their filtration fluxes in Fig. 2. However, when comparing the proportions of $\propto_{b}$ and $\propto_{p}$ for the CV-IOM fractions, it was found that the C-HPI in CV-IOM had the highest proportions of $\propto_{b}$ and $\propto_{p}$, followed by TPI, indicating that the C-HPI and TPI fractions in CV-IOM were more likely to block and constrict pores during UF with both types of IOM, while the N-HPI organics were conducive to the formation of a cake layer due to its highest $\propto_{c}$. A similar phenomenon was also observed in the MA-IOM fractions, of which the C-HPI and TPI organics had higher $\propto_{b}$ and $\propto_{p}$ values than did N-HPI and HPO, whereas the highest $\propto_{c}$ appeared with HPO, suggesting that the HPO in MA-IOM facilitated the formation of cake layer, whereas the membrane fouling via pore blocking and constriction was mainly induced by C-HPI and TPI organics.

### Characterization of membrane surface morphology

**Figure 4:**
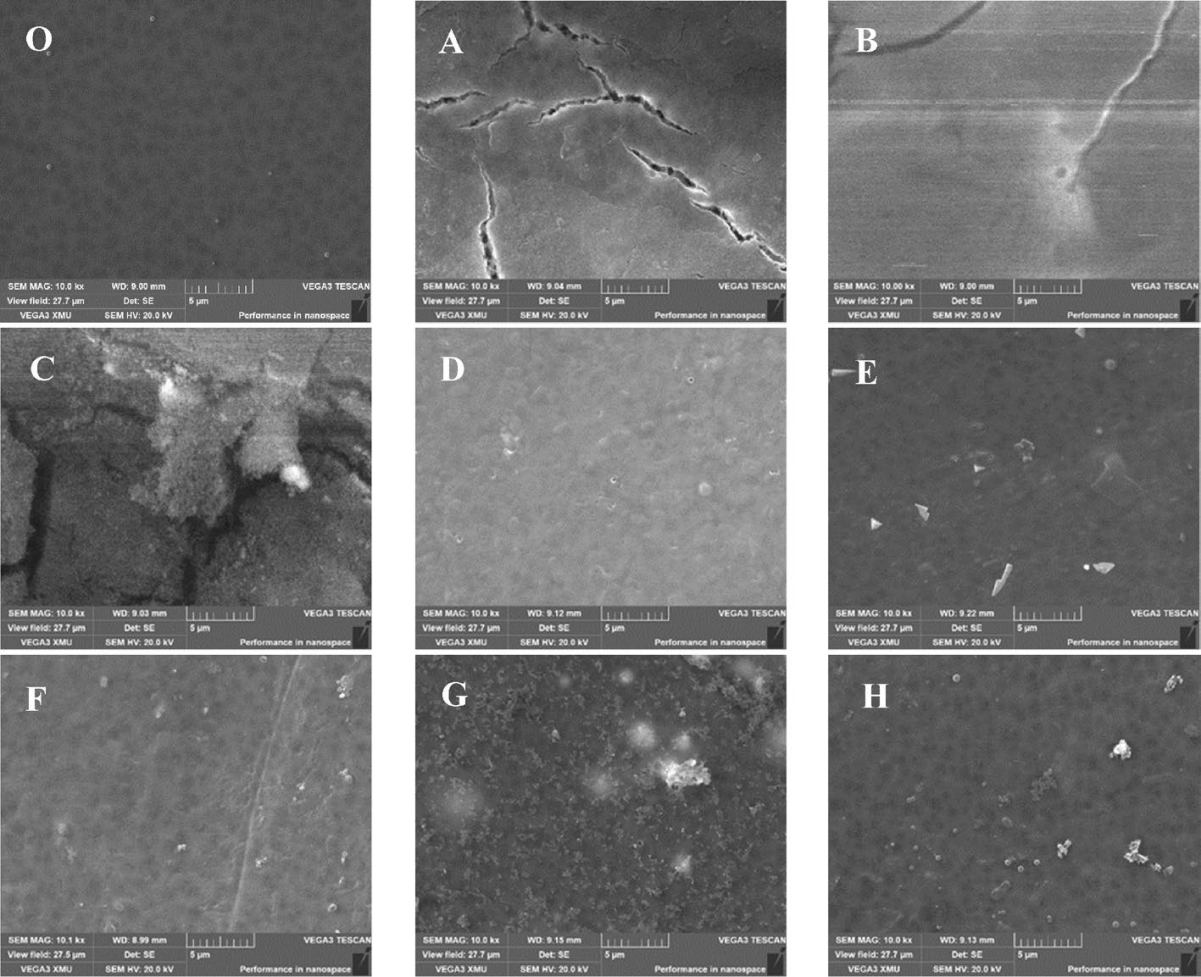
SEM micrograph (bar = 5 μm) of the clean membrane and the fouled membranes under various CV- and MA-IOM fraction filtration treatments, (**O**) clean membrane, (**A**) CV-HPO, (**B**) CV-TPI, (**C**) CV-C-HPI, (**D**) CV-N-HPI, (**E**) MA-HPO, (**F**) MA-TPI, (**G**) MA-C-HPI, (**H**) MA-N-HPI.

Figure 4 shows the surface morphology of the clean and fouled membranes. Membrane was fouled in varying degrees after filtrated by various IOM fractions, and there were some white spots, crystals, or cake layer occurred and accumulated on the membrane surfaces, especially for N-HPI of CV-IOM (D) and HPO of MA-IOM (E) fractions, there were a lot of macromolecular organics accumulated on the membrane surfaces and cake layer formed, which was speculated to contain proteins and polysaccharides or humic-like organics^9^, this result was consistent with the result in Fig. 3. However, when comparing the SEM images of other CV-IOM fractions, it was found that despite the membrane surface was coved with a cake layer by CV-HPO and TPI organics, some gaps and part of unconnected cake layer appeared on the membrane surface, whereas for MA-IOM fractions, disconnected cake layers also appeared in the TPI and C-HPI, which might result in less fouling.

**Figure 5:**
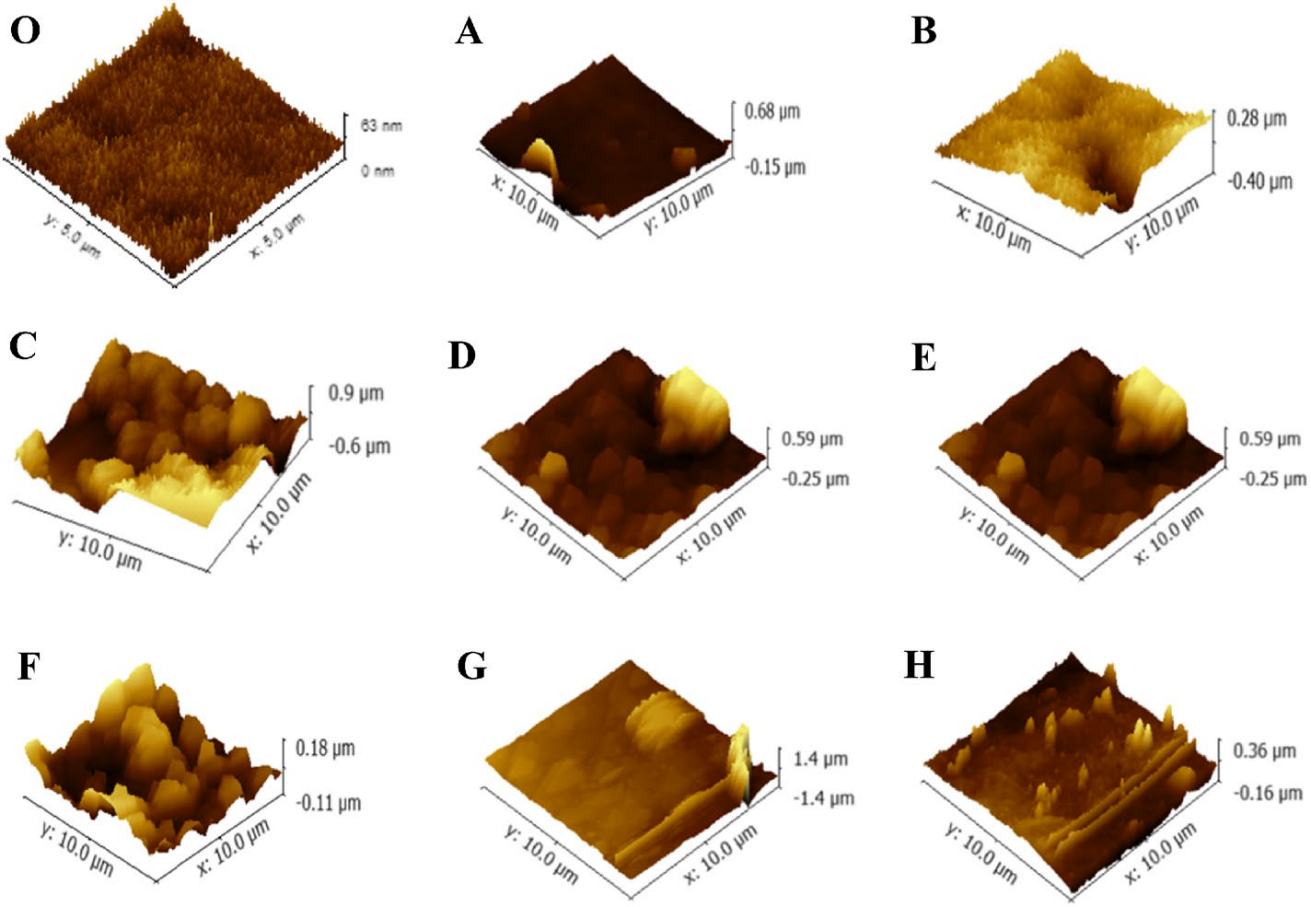
AFM images of the clean and fouled membrane surfaces when filtering various IOM filtrations, (**O**) clean membrane, (**A**) CV-HPO, (**B**) CV-TPI, (**C**) CV-C-HPI, (**D**) CV-N-HPI, (**E**) MA-HPO, (**F**) MA-TPI, (**G**) MA-C-HPI, (**H**) MA-N-HPI.

Figure 5 depicts the AFM images of fouled membranes. The roughness parameters presented as average roughness (R_a_), root-mean-square roughness (R_q_), and peak-to-valley height (R_z_) is also determined (Table 2). The surface topography of the membranes fouled by various IOM fractions reflected by R_a_ were significantly different. The R_a_ was 64.8, 30.9, 0.269, and 90.8 nm for CV-IOM HPO, TPI, C-HPI, and N-HPI, respectively, comparing with 4.092 nm for clean membrane, while they were 75.6, 49.9, 0.232, and 43.8 nm for HPO, TPI, C-HPI and N-HPI of MA-IOM. Similar to R_a_, R_q_, which represents the standard deviation of surface heights, as well as R_z_, were also the highest at N-HPI in CV-IOM and HPO in MA-IOM, suggesting that the membrane fouled by N-HPI of CV-IOM and HPO of MA-IOM had rougher surfaces compared to those fouled by other IOM fractions, which was consistent with their filtration fluxes (Fig. 2). This result further indicated that higher surface roughness might lead to higher fouling risk.

**Table 2:** Quantitative summary of the roughness parameters determined for various fouled membranes.

	R_a_ (nm)	R_q_ (nm)	R_z_ (nm)
Clean membrane	4.092	5.221	63.36
**CA-IOM**			
HPO	30.9	60.1	831.6
TPI	64.8	89.4	681
C-HPI	0.269	0.326	1.523
N-HPI	90.8	136.9	833.9
**MA-IOM**			
HPO	75.6	116.5	800.3
TPI	43.8	58.9	293.8
C-HPI	0.232	0.315	2.814
N-HPI	49.9	59.7	520.2

To better elucidate their discrepancies in membrane fouling evolution, the organic characteristics and variation of the fouling process were analyzed.

### Analysis of the IOM characteristics of various MA-IOM and CV-IOM fractions

Table 3 shows the fractionation results for both types of algal IOM, which were largely (70% or more) hydrophilic. The HPO, TPI, C-HPI, and N-HPI from CV-IOM contained 19.57%, 5.14%, 1.69%, and 73.59% DOC, respectively, whereas those from MA-IOM were 5.74%, 10.77%, 1.95%, and 80%, respectively. These values were similar to the results of Li et al. who found that the N-HPI fraction was the major organic fraction of MA-IOM^14^. Notably, the complex components in the water matrix, i.e., the medium, and the algal species might also lead to a shift in organic hydrophilicity^37^. Although the N-HPI fraction was the major component of the IOM from both algal species, the effect of this fraction on IOM membrane fouling greatly differed between the species, which indicated that the IOM filtration flux might be associated with not only the hydrophobicity but also the composition of organics, as well as the average hydrodynamic radius of the organic matter^38^.

**Table 3:** Hydrophilic properties of CV-IOM and MA-IOM.

	HPO (%)	TPI (%)	C-HPI (%)	N-HPI (%)
CV-IOM	19.57 ± 0.02	5.14 ± 0.05	1.69 ± 0	73.59 ± 0.04
MA-IOM	5.74 ± 0.01	10.77 ± 0	1.95 ± 0	80 ± 0.09

**Figure 6:**
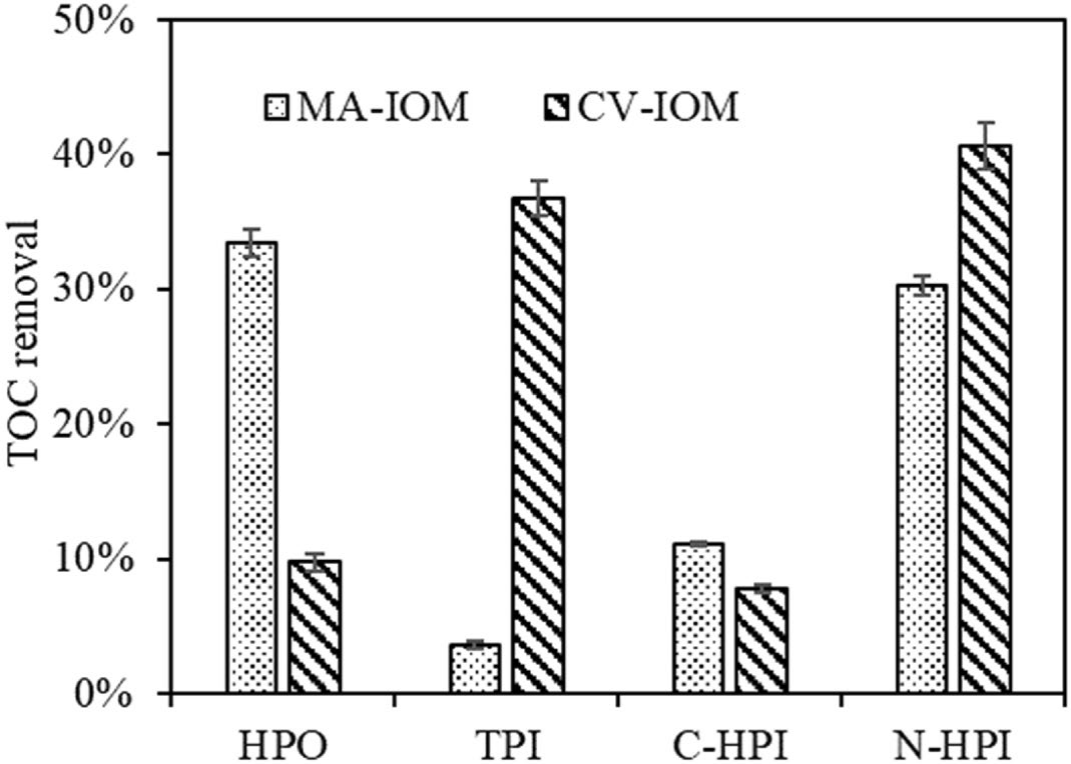
Organic removal of various CV-IOM and MA-IOM fractions.

Figure 6 presents the effectiveness of organic removal. Regardless of the algal species, the N-HPI fraction was significantly reduced by UF, and the removal efficiency was 30% and 40% for MA-IOM and CV-IOM, respectively. Moreover, the HPO organics in MA-IOM and the N-TPI fraction in CV-IOM were clearly rejected, suggesting that HPO and N-HPI in MA-IOM and N-HPI and TPI in CV-IOM were the main materials that induced IOM membrane fouling, which was in accordance with their filtration fluxes.

**Figure 7:**
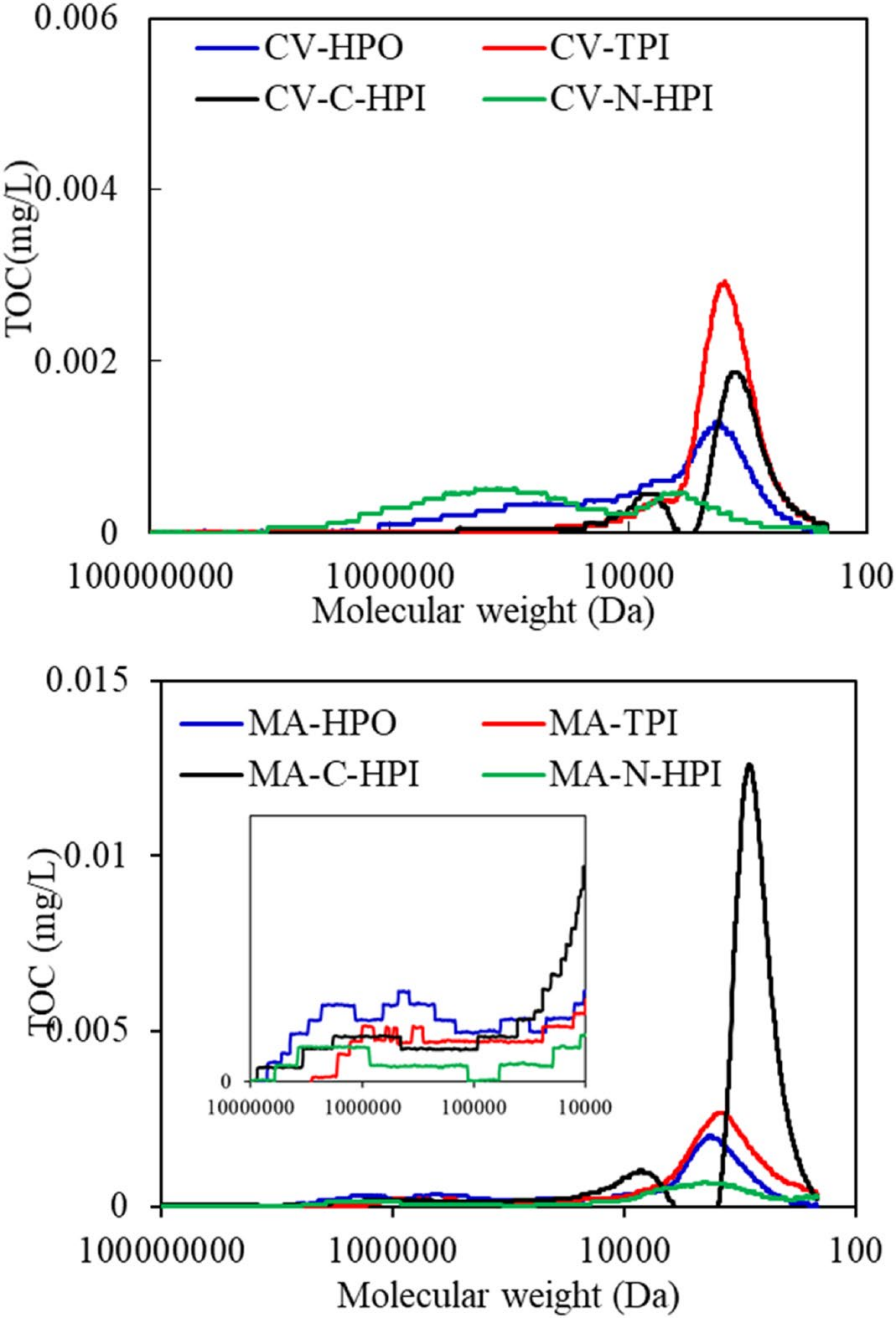
MW distributions of CV-IOM and MA-IOM fractions (DOC 5 ± 0.05 mg/L).

The MW distributions of the IOM fractions were also determined (Fig. 7). Generally, the MWs of organics in both IOMs can be divided into three peaks: peak A (1,000 K Da) was related to biopolymers (BP, such as polysaccharides or amino sugars), peak B (6,500 Da) was related to humic-like substances (HS), and peak C corresponded to (1,200 Da) building blocks of low-MW acids and humics (LMWA&BB)^35,39^. N-HPI and HPO produced the highest peak areas for macromolecular substances in CV-IOM, while the peak A areas were mainly associated with HPO in MA-IOM, indicating that the N-HPI and HPO fractions were the major components of the macro-MW organics in CV-IOM, but the macro-MW substances in MA-IOM were mainly consisted of the HPO fraction. A high proportion of N-HPI organics, which was reported to consist of biopolymers^40^, would reduce the water treatment efficiency, because such organics are less readily reduced by conventional coagulation, sedimentation, and filtration than hydrophobic fractions, which again indicated the problem caused by IOM during treatment of algae-containing water. However, the peak areas of the MA-IOM fractions varied significantly. The HPO fractions had the highest macro-MW organics over 10 kDa, followed by N-HPI, TPI, and C-HPI, consistent with the filtration fluxes in Fig. 2.

In addition, macromolecular substances were found to significantly decrease after UF (Fig. S1), which verified the assumption that macromolecular substances were the major organics that caused UF fouling, for MA-IOM, macro MW organics of HPO fraction induced the major membrane fouling, whereas for CV-IOM, it was the macro MW organics of N-HPI fraction that caused the major UF fouling.

### Discussion of membrane fouling on the basis of the XDLVO theory

#### Analysis of the properties of the membrane and foulants

Generally, the surface properties of membranes and foulants, i.e., surface charge and hydrophobicity, significantly influence membrane fouling. Table 4 displays the contact angles for the membrane and foulants. The clean membrane had a low water contact angle of 58° (< 90°), which indicated a hydrophilic surface. The water contact angles of the IOM fraction components after UF changed significantly. For example, the contact angles of N-HPI and HPO in both IOMs increased, while those of TPI and C-HPI significantly decreased. The N-HPI and HPO fractions in both IOMs had higher contact angles than the TPI and C-HPI fractions, indicating that after UF of IOM containing HPO and N-HPI materials, the cake layers formed on the membrane surfaces might be predominantly hydrophobic. The hydrophobicity of N-HPI based on the water contact angle can be associated with its polymeric polysaccharides, such as amino sugars, chitin-like organics, and high-nitrogen compounds, yet the hydrophobicity of HPO should be related to its aromatic groups, as indicated by prior researches^41^. TPI and C-HPI had lower water contact angles, indicating that TPI and C-HPI in both IOMs might have more hydrophilic effects on the membrane. Notably, a high water contact angle might also reflect nanoscale changes in the roughness of membrane surfaces, such as the “lotus leaf-effect”, as suggested by prior studies^37^ and Fig. 4.

**Table 4:** Properties of the membrane and IOM fractions.

	Contact angle (°)			Zeta potential (mV)	Average radius (nm)
	θ_wat_	θ_gly_	θ_dii_		
Clean	54.65 ± 1.14	60.242	26.23 ± 0.645	− 17 ± 0.02	/
**CV-AOM**					
HPO	75.41 ± 1.24	80.9 ± 0.42	35.22 ± 0.35	− 10.15 ± 0.45	647.6 ± 3.53
TPI	19.13 ± 1.15	36.48 ± 0.88	13 ± 0.26	− 14.47 ± 0.72	267.4 ± 1.31
C-HPI	22.8 ± 2.01	38.32 ± 0.59	17.93 ± 0.15	− 23.53 ± 0.18	1362 ± 1.21
N-HPI	85.2 ± 1.48	81.51 ± 1.13	43.95 ± 0.66	− 21.7 ± 0.431	640.4 ± 0.42
**MA-AOM**					
HPO	86.7 ± 1.21	74.14 ± 1.70	30.69 ± 0.89	− 14.6 ± 0.52	326 ± 0.53
TPI	68.38 ± 0.84	57.43 ± 0.46	29.41 ± 0.84	− 3.18 ± 0.24	664.3 ± 1.11
C-HPI	77.63 ± 0.75	80.61 ± 0.77	45.08 ± 0.95	4.05 ± 0.07	436.5 ± 0.36
N-HPI	86.55 ± 1.44	80.46 ± 2.08	40.13 ± 0.66	0.17 ± 0.21	739.1 ± 5.99

Contact angle values are given as mean value ± standard deviation (n = 10), zeta potential and average radius values are given as mean value ± standard deviation (n = 3).

The variation in zeta potential and average radius revealed that the C-HPI in CV-IOM possessed the smallest zeta value, followed by the N-HPI, TPI, and HPO in CV-IOM, while the lowest zeta values occurred in HPO of MA-IOM, signifying differences in the colloidal stability. Nevertheless, as all the CV- and MA-IOM fractions possessed a mean hydrodynamic radium over 100 nm, all can be regarded as colloidal^38^.

#### Cohesion free energy of the membrane and foulants

Table S1 displays the surface tension components for the clean membrane and foulants. The clean membrane exhibited high γ^LW^ and electron-donor monopolarity ($\gamma_{{}}^{ - }$) but low electron-acceptor components ($\gamma_{{}}^{ + }$), illustrating that the membrane used in this study might have polar properties. This observation was in line with prior research showing that polymeric membranes are commonly characterized by a high $\gamma_{{}}^{ - }$^27,31^. Previous studies indicated that surfaces with significant electron-acceptor capacity ($\gamma_{{}}^{ + }$) would interact favorably with surfaces that possess electron-donor functionality, thus inducing potentially attractive AB interactions^27^. The HPO and N-HPI of CV-IOM had high $\gamma_{{}}^{ - }$ and $\gamma_{{}}^{ + }$ values, corresponding to strong AB interactions. However, the HPO in MA-IOM had a relatively low $\gamma_{{}}^{ + }$, and the N-HPI in MA-IOM had a relatively low $\gamma_{{}}^{ - }$; therefore, their AB interactions might be weaker. The N-HPI in CV-IOM had the highest r^AB^, suggesting that the chemical bonds among N-HPI organic molecules in CV-IOM were the strongest; thus, the cake layer formed during UF of CV-IOM might be the densest.

Table 5 presents the cohesion free energy ($\Delta G^{CO}$) of the membrane and foulants $\Delta G^{CO}$ is the interaction energy when two surfaces of the same material are immersed in water and brought into contact and can provide quantitative insight into the thermodynamic stability (hydrophobicity or hydrophilicity) of solid surfaces^26^. From the $\Delta G^{CO}$ data for the CV-IOM and MA-IOM fractions, it was found that $\Delta G^{CO}$ changed significantly among the various IOM fractions. Almost all the $\Delta G^{CO}$ values were negative for all the IOM fractions, except for TPI in CV-IOM and C-HPI in MA-IOM. Generally, a positive $\Delta G^{CO}$ value implies a hydrophilic surface, whereas a negative $\Delta G^{CO}$ value suggests a hydrophobic surface. The above result thus indicated that the TPI in CV-IOM and C-HPI in MA-IOM were more hydrophilic than the other fractions, which might be associated with the discrepancies in their organic components, molecular structures, and functional groups. The most negative $\Delta G^{CO}$ was found for the N-HPI fractions of both IOMs, followed by the HPO fractions, suggesting that after UF, the cake layer formed by the N-HPI fractions in both IOMs might be more hydrophobic and denser than that formed by the other fractions. It was of note that although the N-HPI in MA-IOM had the most negative $\Delta G^{CO}$, the r^AB^ was not large (Table S1); therefore, when the N-HPI in the MA-IOM component was likely to aggregate together, because the chemical bonds among the various N-HPI organic molecules in the MA-IOM fraction were not strong, and the content of macro MW organics was not very high (Fig. 7), the cake layer formed might not be dense, whereas the N-HPI in CV-IOM might have formed a denser cake layer, which was in accordance with their UF fluxes and SEM images in Figs. 2 and 4 .

**Table 5:** Adhesion free energy between the membrane and foulants upon contact and cohesion free energy between membrane and foulants.

	$\Delta G^{AD}$				$\Delta G^{CO}$			
	$\Delta G^{LW}$	$\Delta G^{AB}$	$\Delta G^{EL}$	$\Delta G^{AD}$	$\Delta G^{LW}$	$\Delta G^{AB}$	$\Delta G^{EL}$	$\Delta G^{CO}$
**CV-IOM**								
HPO	3.587	4.131	− 0.059	7.658	− 32.627	15.000	0.023	− 17.604
TPI	− 1.767	2.635	0.042	0.909	− 7.922	58.689	0.047	50.815
C-HPI	− 1.052	3.994	0.000	2.943	− 2.808	− 5.035	0.124	− 7.719
N-HPI	− 2.332	8.131	0.036	5.835	− 13.798	− 25.155	0.105	− 38.848
**MA-IOM**								
HPO	− 1.337	− 5.022	0.043	− 6.315	− 4.531	− 22.078	0.048	− 26.561
TPI	2.406	1.112	− 0.386	3.132	− 14.685	13.822	0.002	− 0.861
C-HPI	− 0.533	− 0.213	− 0.939	− 1.685	− 0.721	4.265	0.004	3.547
N-HPI	3.420	− 2.371	− 0.615	0.434	− 29.675	− 5.784	0.000	− 35.459

#### Adhesion free energy of the membrane and foulants

The adhesion free energies ($\Delta G^{AD}$) per unit area between the membrane and foulants were also calculated, and the data can be found in Table 5. The adhesion free energy ($\Delta G^{AD}$) is the interaction energy between the membrane and foulants. From the calculated $\Delta G^{AD}$ at $h_{0}^{{}}$ of 0.158 nm, it can be found that all the $\Delta G^{AD}$ values, except for those associated with HPO and C-HPI in MA-IOM, were positive. These values indicated that the IOM fractions of CV and MA (except for HPO and C-HPI) underwent repulsive interactions upon contact with the membrane, whereas stronger attractive interactions might have occurred between the membrane and HPO and C-HPI of MA-IOM. Thus, the HPO and C-HPI organics of MA-IOM were easily intercepted by the membrane or inside the membrane pores, which might have led to high irreversible fouling. Li et al. reported that $\Delta G^{AD}$ can be closely related to the decline in the initial filtration flux^42^. The $\Delta G^{AD}$ values for the HPO in MA-IOM was obviously more negative than those for the other fractions, suggesting that when HPO in MA-IOM gained access to the membrane surface, the attractive interaction was the highest; thus, the membrane might have been the most easily fouled by the HPO in MA-IOM, which explains its effect on the filtration flux.

#### Interfacial energy between the membrane and foulants on a clean membrane surface

**Figure 8:**
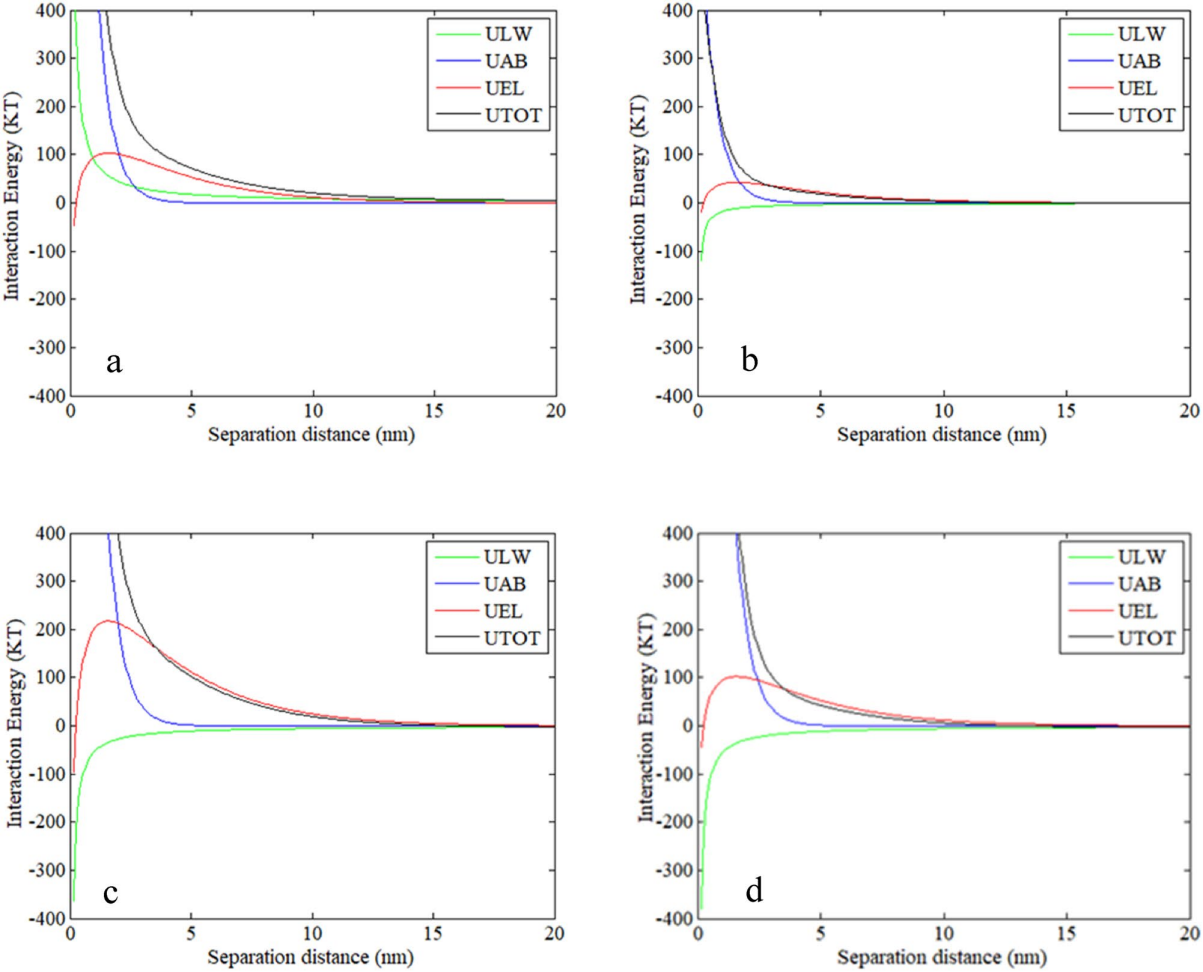
Interaction energy profiles between clean membrane surface membrane and CV-IOM foulants using the XDLVO theory as a function of separation distance, (**a**) HPO, (**b**) TPI, (**c**) C-HPI, (**d**) N-HPI.

To ascertain the interaction energy between the membrane and IOM fractions, the interfacial energy profiles between the clean membrane surface and foulants were calculated. Figure 8 displays the interaction energy profiles between the clean membrane surface and CV-IOM foulants. From Fig. 8, it can be seen that the AB energy of all the fractions was repulsive and exerted important effects at a short distance of < 5 nm, yet the EL energy of all the CV-IOM fractions was repulsive over a long distance of < 15 nm, which might be explained by the negatively charged surface and thick electrical double layer in this system. For LW, the energy was attractive except with HPO, which could be explained by the hydrophilic nature of the HPO organics in CV-IOM (88.49 mJ/m^2^). The integration of the results for the AB, EL, and LW components illustrates the organic-membrane interactions when the CV-IOM fractions nearing the membrane surface, constituted membrane fouling. Figure 8 shows that all the CV-IOM fractions were subjected to repulsive interactions when approaching the membrane surface at 12 nm. Then, the IOM components encountered enhanced electrostatic repulsion interaction before reaching to the membrane surface. The energy barriers were 31.5, 23.6, 65, and 37.65 kT for HPO, TPI, C-HPI, and N-HPI of CV-IOM, respectively, indicating that the membrane had more repulsive interactions with the C-HPI and N-HPI fractions, but the TPI fraction may be initially deposited due to its lower energy barrier. In addition, after all four fractions exceeded the greatest energy barrier, the LW and AB components played crucial roles in the membrane-foulant interaction after counteracting or reinforcing part of the repulsive interaction, which was beneficial for the adsorption of the IOM foulants. As the LW and AB energy components had large magnitudes at < 3 nm, the total maximum interactions were repulsive. The total maximum interactions between the membrane and CV-IOM fractions were 2780.2 kT, 450.45 kT, 4083 kT, and 3923.9 kT for HPO, TPI, C-HPI, and N-HPI, respectively (Fig. S4). TPI and HPO had lower repulsion interactions than the other fractions, indicating that when TPI and HPO exceeded the maximum energy barriers, the TPI and HPO organics in CV-IOM contaminated the membrane to a more severe extent during the initial filtration flux due to their lower repulsion interactions.

**Figure 9:**
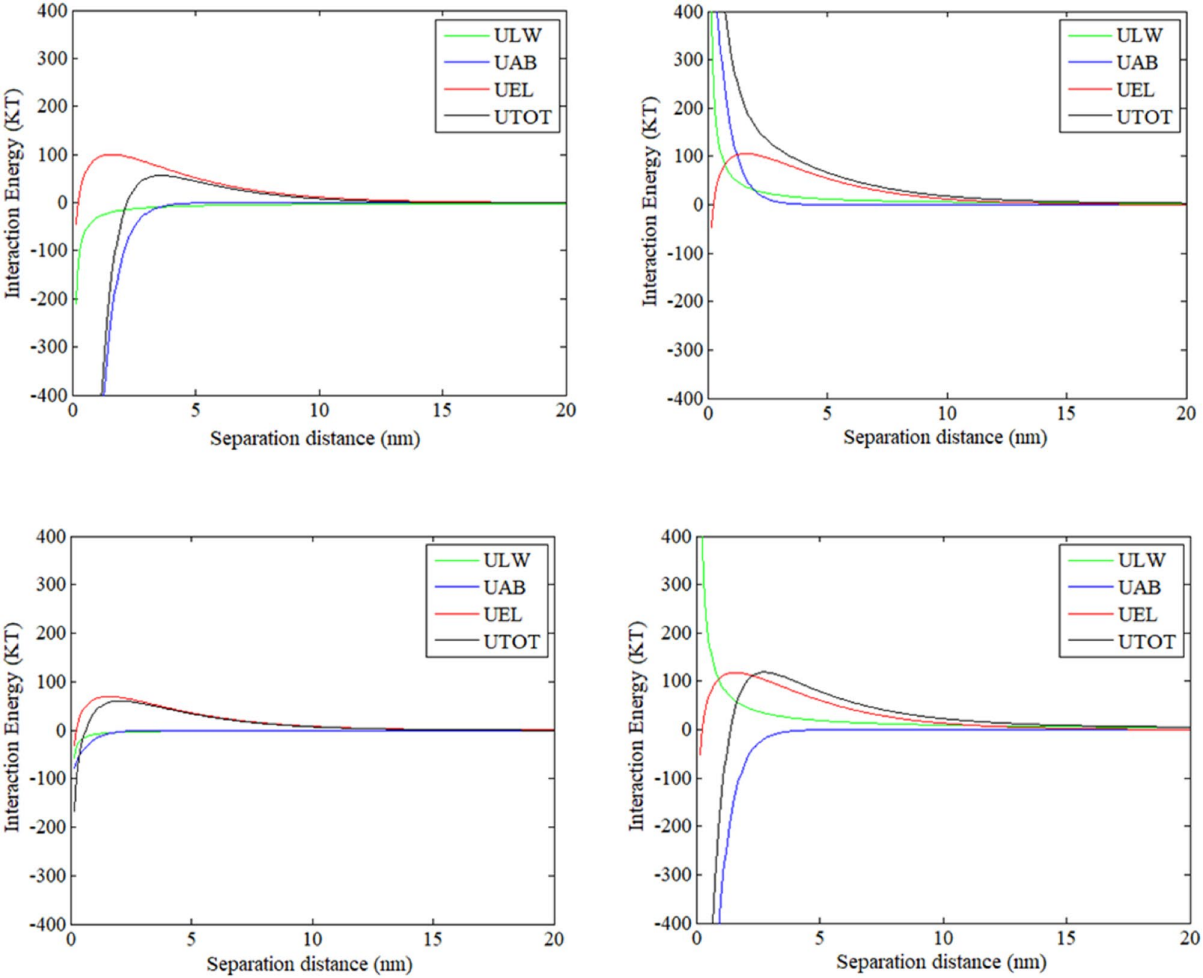
Interaction energy profiles between the clean membrane surface membrane and MA-IOM foulants using the XDLVO theory as a function of separation distance, (**a**) HPO, (**b**) TPI, (**c**) C-HPI, (**d**) N-HPI.

The profiles of the interfacial energy between the MA-IOM fractions and the clean membrane surface (Fig. 9) revealed that all the MA-IOM fractions experienced repulsion at a distance of 20 nm and achieved maximum repulsion at 12 nm. The maximum energies for the MA-IOM fractions were 56.6, 50, 62, and 125 kT for HPO, TPI, C-HPI, and N-HPI of MA-IOM, respectively, suggesting that when the MA-IOM components reached the membrane surface via the permeate flow near the membrane surface, the HPO and TPI fractions had lower repulsive interaction energies to exceed than did the N-HPI and C-HPI fractions. Additionally, after the fractions exceeded the highest energy barrier at < 5 nm, the total maximum interactions between the membrane and the MA-IOM HPO, TPI, C-HPI, and N-HPI fractions were − 2890, 969.5, − 166.66, and − 884.1 kT, respectively. Therefore, after the MA-IOM fractions exceeded the energy barriers, the HPO organics, followed by the N-HPI fraction, in MA-IOM contaminated the membrane to the greatest extent during the initial filtration flux, which agreed with the filtration flux results. Of note, as the primary filtration process might be very fast, the rate and extent of fouling would be determined by the interaction between bulk foulant molecules and foulant molecules deposited in the fouling layer^42^.

#### Interaction energy profiles between IOM/EOM fractions and the fouled membrane surface

The primary adherence of colloids to the membrane surface could be determined by the total interaction between the membrane and foulants when the foulants approach the membrane surface. Over time, the membrane surface becomes covered with foulants, and fouling tendencies are governed by the cohesion energy between new foulants and foulants on the fouled membrane surfaces^22^.

**Figure 10:**
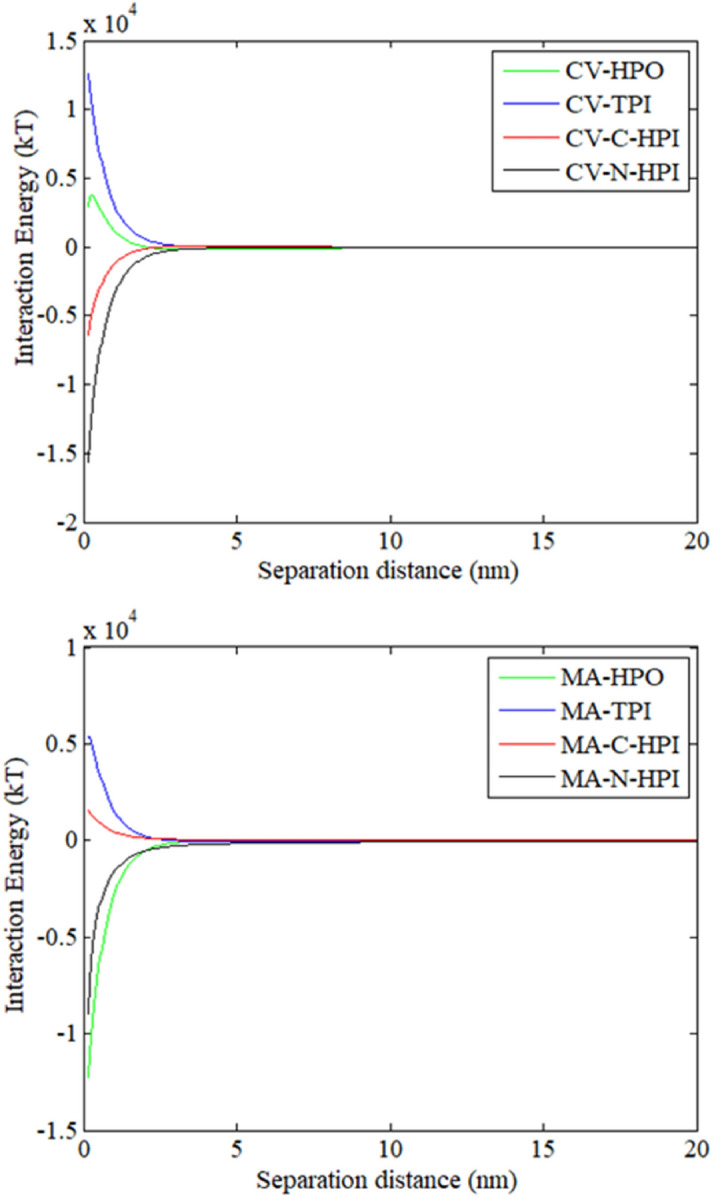
Interaction energy profiles between the fouled membrane surface membrane and CV-IOM and MA-IOM fractions.

Figure 10 depicts the profiles of the interaction energy between new foulants and foulants on the membrane surface. The CV-IOM HPO and N-HPI fractions experienced weak attraction at a distance of > 12 nm, while the C-HPI and TPI fractions encountered weak repulsion at a distance of > 12 nm owing to the presence of AB repulsion and LW attraction. When the CV-IOM fractions contacted the fouled membrane surface, the energy barriers were − 118.5, 2, 102, and − 16.1 kT for HPO, TPI, C-HPI, and N-HPI, respectively, indicating that the N-HPI and HPO fractions of CV-IOM experienced higher adsorption energies than the TPI and C-HPI fractions did, whereas the C-HPI and/or TPI fractions had to exceed stronger repulsive interaction energies to approach to the fouled membrane. Therefore, when the C-HPI and/or TPI fractions of CV-IOM came close to the fouled membrane surface, the membrane fouling rate may have been lower than that of the N-HPI and/or HPO interactions. However, at a separation distance of < 5 nm, the AB attraction predominated, and the maximum energies were 2800, 12,560, − 6780, and − 15,708 kT for HPO, TPI, and C-HPI, and N-HPI of CV-IOM, respectively. The N-HPI foulants had the highest attractive energies with the fouled membranes, which was consistent with the filtration flux. Notably, the manner in which the energy varied over time could also be determined from other physicochemical parameters, such as the particle concentration, applied pressure (which governs the permeation drag), cross-flow velocity, electrolyte concentration, as well as hydration force and calcium ion complexation^27^.

For MA-IOM, the maximum interaction energies were 27.5, − 24.5, 5, and − 100 kT for HPO, TPI, C-HPI, and N-HPI of MA-IOM, respectively, which indicated that when MA-IOM HPO and/or C-HPI approached the contaminated membrane surfaces, HPO and/or C-HPI had to exceed larger repulsive interaction energies than did N-HPI and/or TPI. However, at a separation distance of < 5 nm, the AB attraction predominated, and the maximum energies were − 12,340, 5180, 1445, and − 9104 kT for MA-IOM HPO, TPI, C-HPI, and N-HPI, respectively, which was in line with their filtration fluxes. Notably, despite the great discrepancies among the interaction energies of the four fractions, the profiles of the interaction energy between the foulants and fouled membrane surfaces were significantly higher than those between foulants and cleaned membrane surfaces, which suggested that the interaction energy between the membrane and foulants in the later filtration process was the main factor determining IOM membrane fouling.

Combining these data with the interaction energies calculated between foulants and clean/fouled membrane surfaces and membrane fouling behaviors, the authors argue that pore blocking might be the dominant membrane fouling mechanism for CV-IOM during the initial filtration flux, yet with increasing filtration time, the membrane fouling might be governed by a cake-enhanced mechanism for both IOM solutions. The resistance of the cake layer might exert a more significant effect than pore blocking during both CV-IOM and MA-IOM UF, organics characteristics combined with free energy analysis from microscopic perspective was suggested for the better understanding of membrane fouling mechanisms.

## Conclusion

UF membrane fouling and the associated mechanism caused by the IOM released from CV and MA were investigated, and the following conclusions can be drawn.

1. Both CV- and MA-IOM caused severe membrane fouling during treatment of algae-containing water; however, the membrane fouling caused by MA-IOM was very different from that caused by CV-IOM. N-HPI was the organic material that caused the most severe membrane fouling during CV-IOM filtration, whereas MA-IOM membrane fouling was mainly caused by HPO organics.
2. From analysis based on the XDLVO theory, it was found that the interaction energy between the membrane and foulants during the later stage of filtration was the main factor determining both the CV-IOM and MA-IOM membrane fouling. The TPI organics in CV-IOM were proposed to foul the membrane to a more severe extent than the other materials during the initial filtration flux; however, when the membrane surface was covered with CV-IOM foulants, the N-HPI fraction of CV-IOM was speculated to cause the most severe membrane fouling due to its highest attractive energy with the fouled membrane.
3. The HPO organics in MA-IOM were found to foul the membrane to the greatest extent during the initial filtration flux, followed by N-HPI. However, when the membrane surface was covered with MA-IOM, the HPO organics were found to foul the membrane to the greatest extent because of their greatest attractive energy with the fouled membrane, followed by N-HPI.
4. From the analysis of modified filtration models, it was found that cake layer formation played an important role during the UF of both CV-IOM and MA-IOM.

## Supplementary Information

Supplementary Information 1.
