# Supplementary material for: Investigation of membrane fouling mechanism of intracellular organic matter during ultrafiltration
Source: Sci Rep. 2021 Jan 13;11:1012. doi: 10.1038/s41598-020-79272-4 (PMC7806927; doi:10.1038/s41598-020-79272-4)
Supplement: Supplementary file 1 — Supplementary Information 1. [file 41598_2020_79272_MOESM1_ESM.docx]

Investigation of membrane fouling mechanism of intracellular organic matter during ultrafiltration

Weiwei Huang^a,c^, Yuanhong Zhu^c^, Bingzhi Dong^b^, Weiwei Lv^a^, Quan Yuan^a^, Wenzong Zhou^a*^, Weiguang Lv^a*^

a Eco-Environmental Protection Research Institute, Shanghai Academy of Agricultural Sciences, Shanghai 201403, China
b School of Environmental Science and Engineering, Tongji University, Shanghai 200092, China

c Shanghai Qingpu Modern Agriculture Park, Shanghai 201403, China

^*^Corresponding author: Tel. +86 21 62208660; fax: +86 21 62208660.
E-mail: lovecity@outlook.com

**List of supplementary material:**

| **Material** | **Page** |
| --- | --- |
| FIGURE S1 MW distribution of IOM after UF, a MA-IOM fraction, b CV-IOM fraction  FIGURE S2 Linear regression equations according to three fouling models of various CV-IOM fractions, a HPO, b TPI, c C-HPI, d N-HPI  FIGURE S3 Linear regression equations according to three fouling models of various MA-IOM fractions, a HPO, b TPI, c C-HPI, d N-HPI  FIGURE S4 Interaction energy profiles between the clean membrane surface membrane and CV-IOM and MA-IOM fractions  TABLE S1 Surface tension components of clean membrane and foulants | S3  S4  S5  S6  S7 |


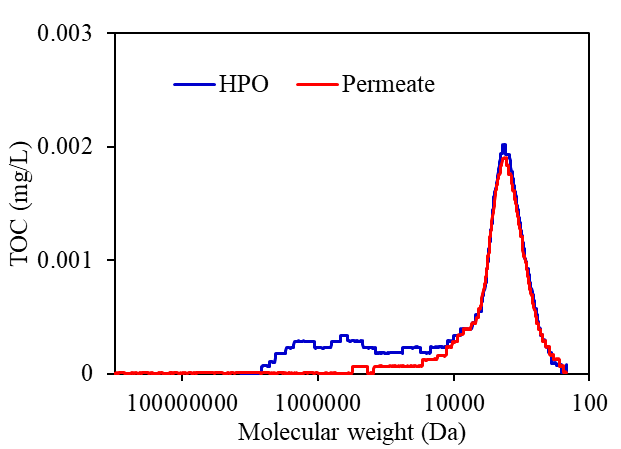

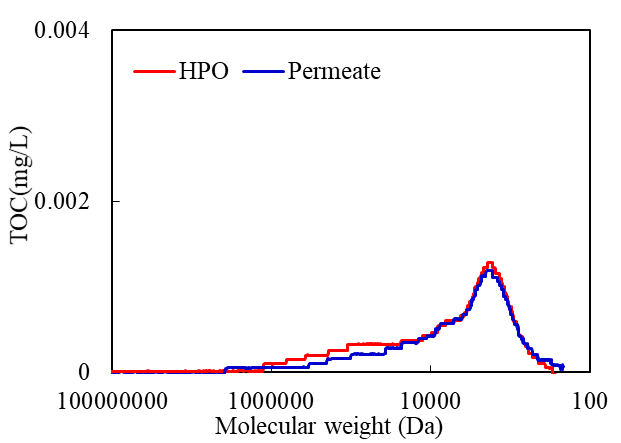


a

b


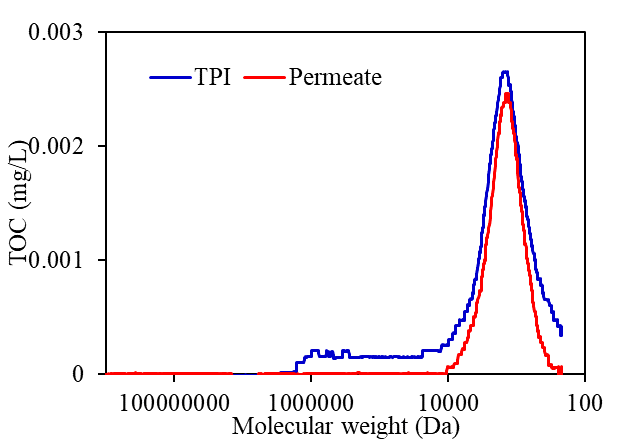

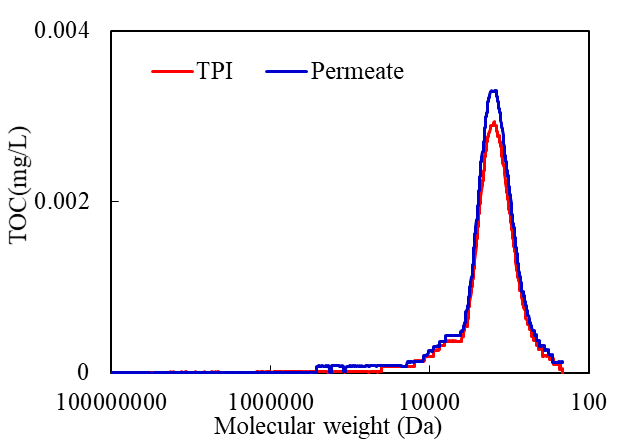


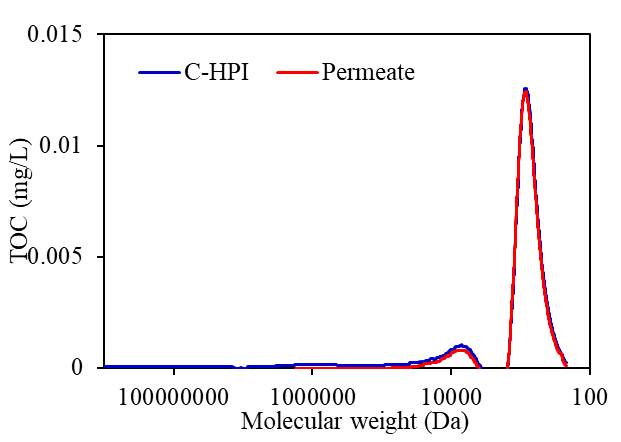

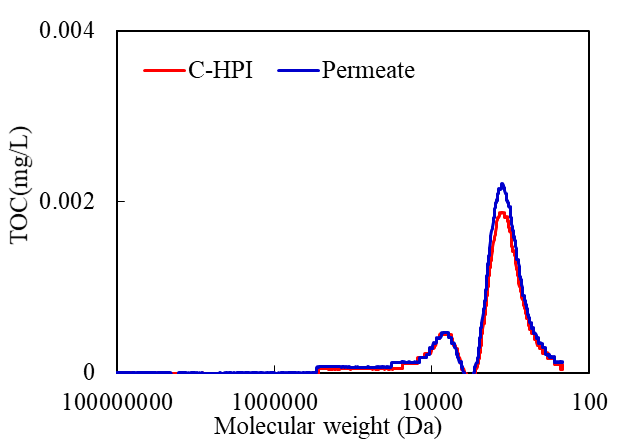


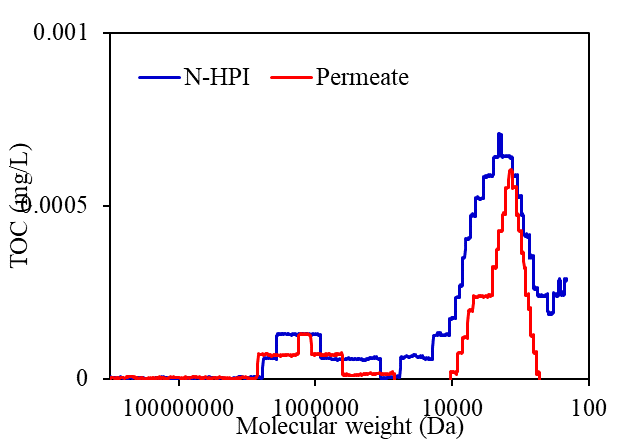

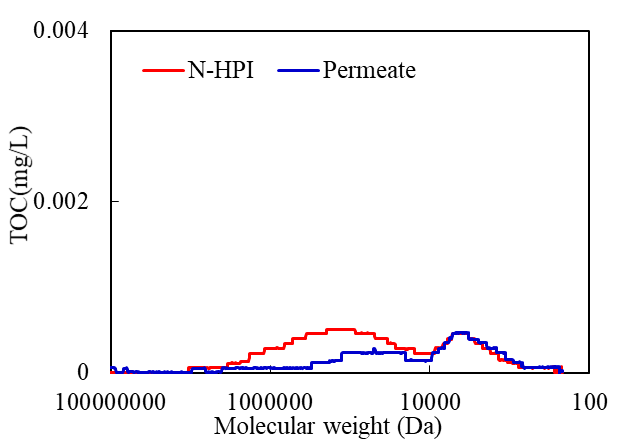


Fig.S1. MW distribution of IOM after UF, a MA-IOM fraction, b CV-IOM fraction

a


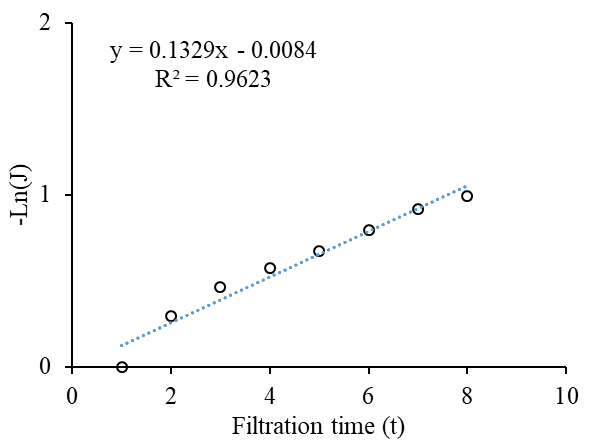

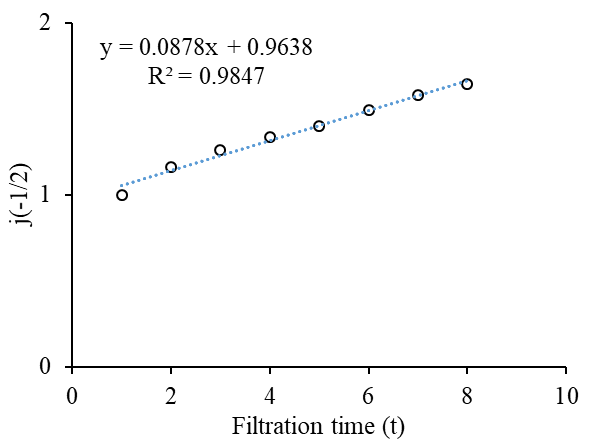

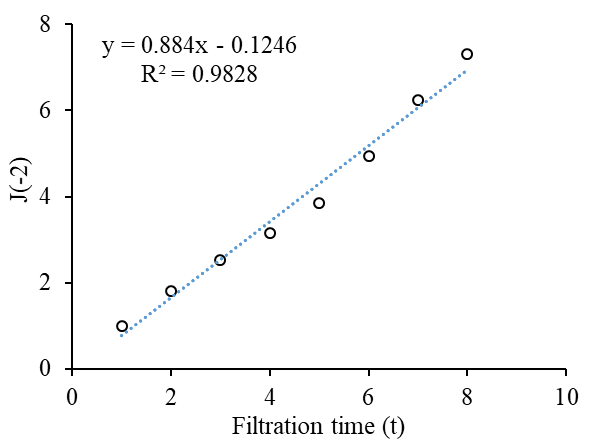


b


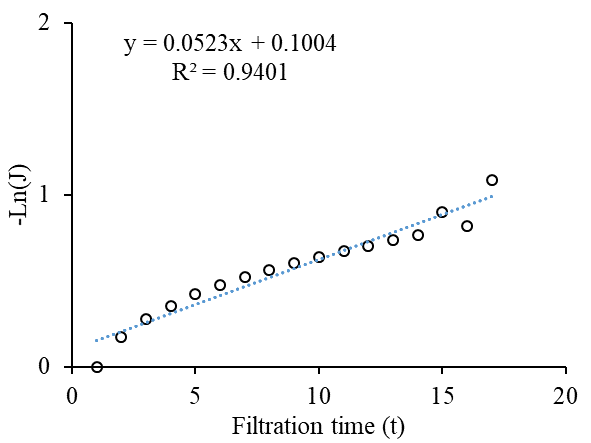

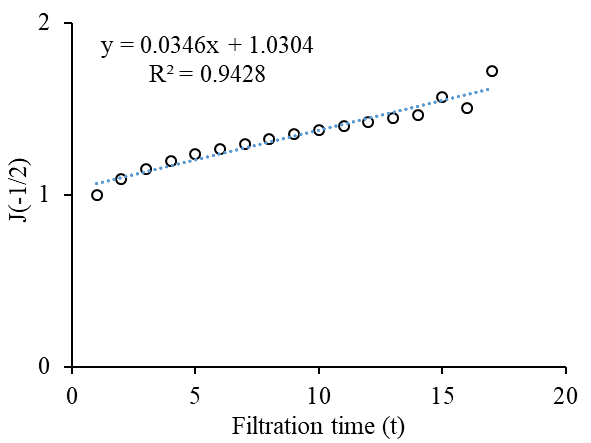

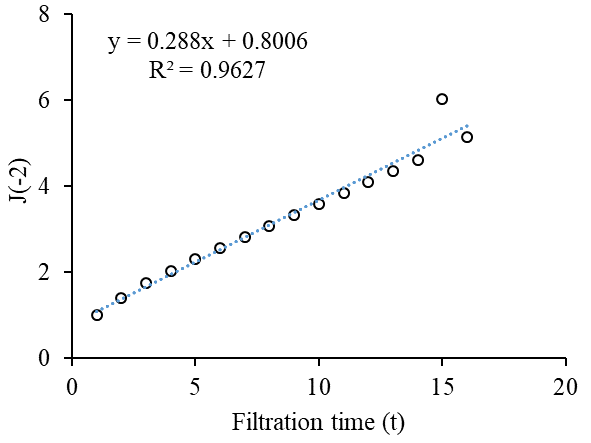


c


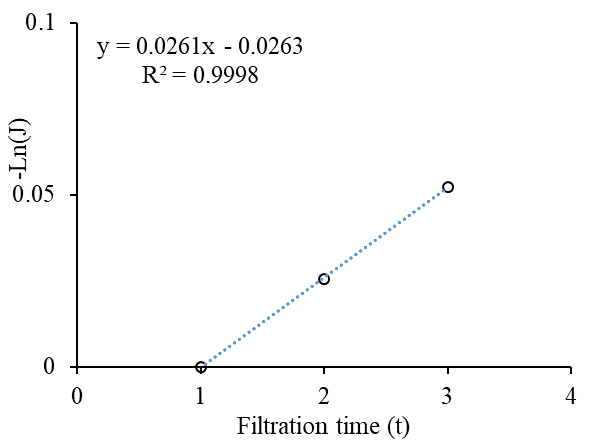

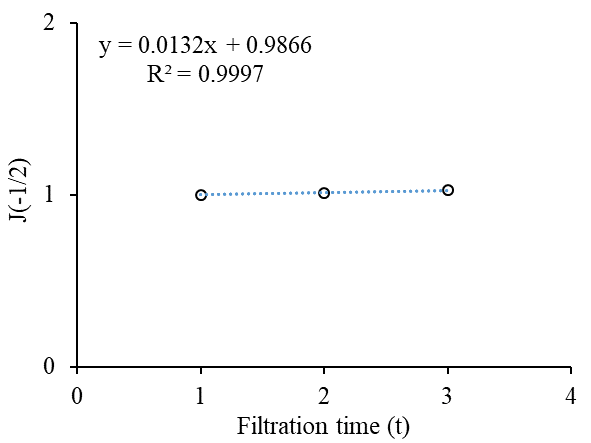

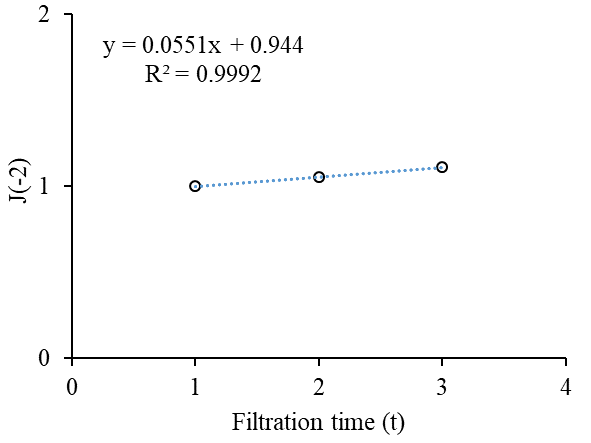


d


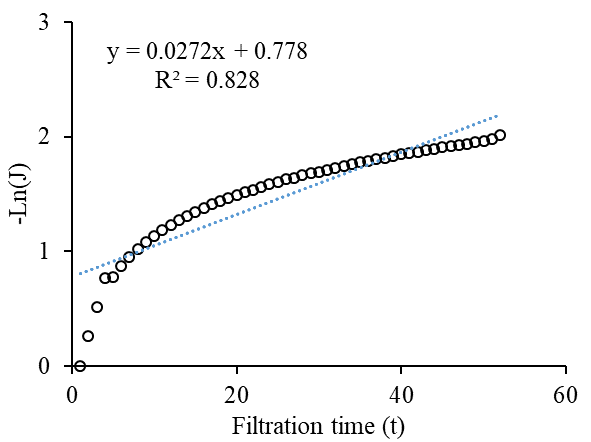

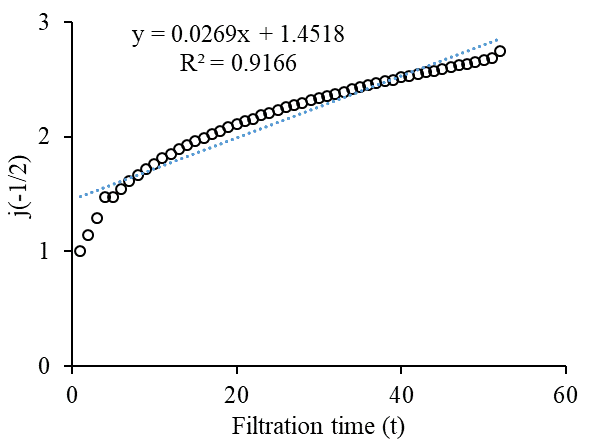

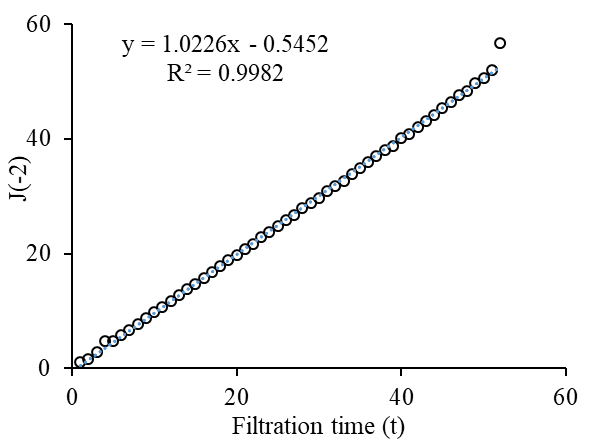


Fig. S2 Linear regression equations according to three fouling models of various CV-IOM fractions, a HPO, b TPI, c C-HPI, d N-HPI


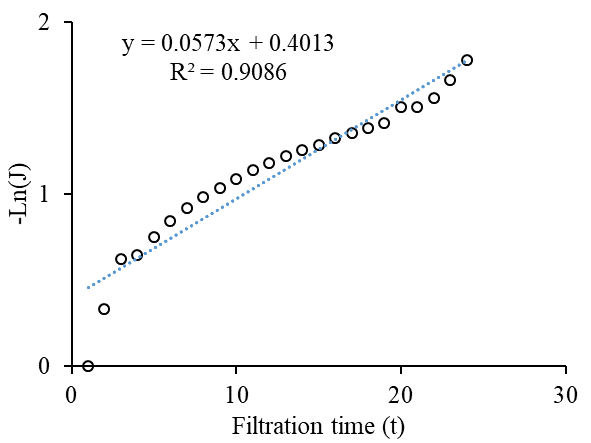

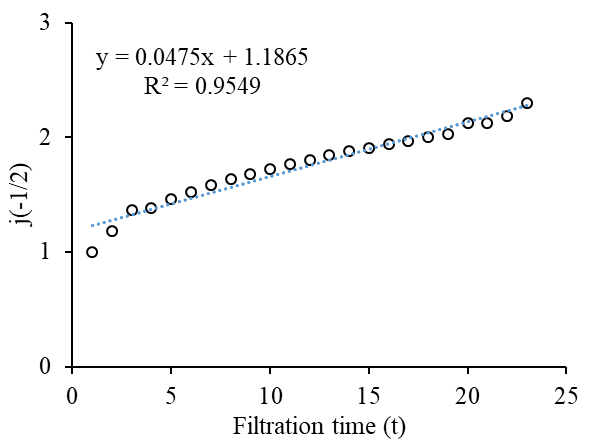

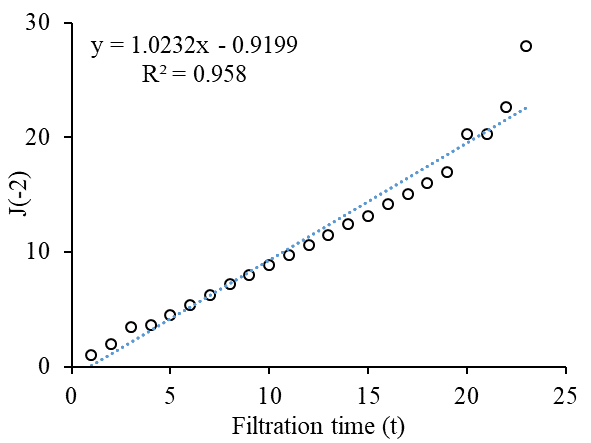


a

b


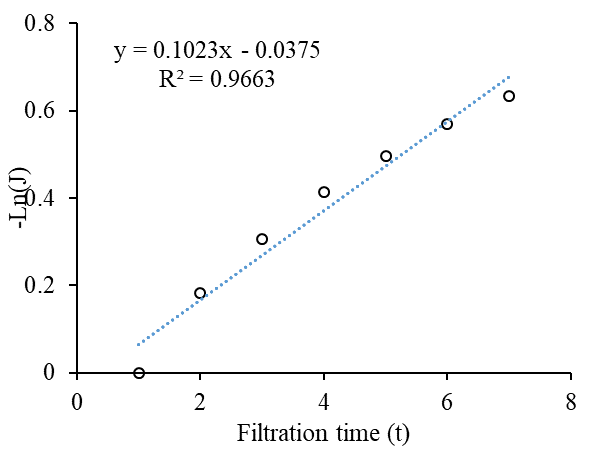

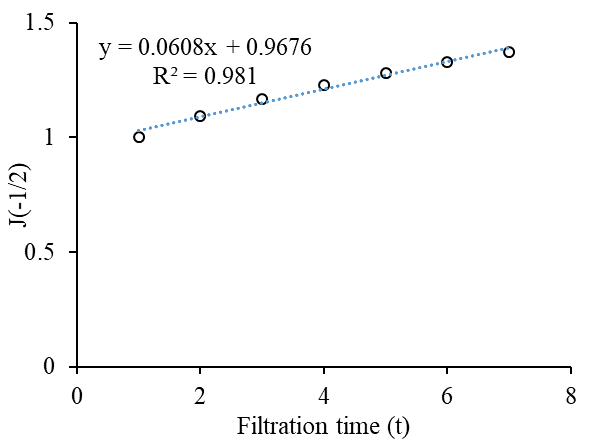

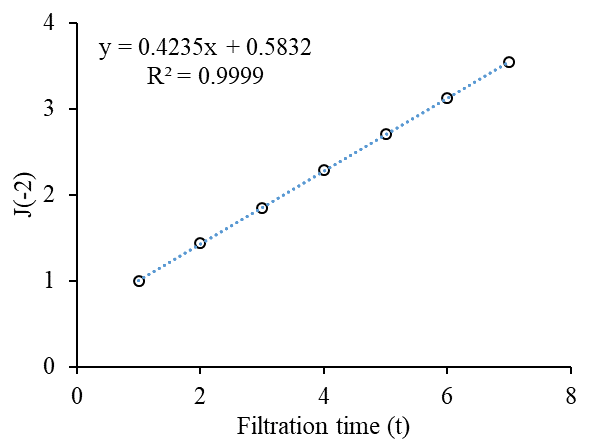


c


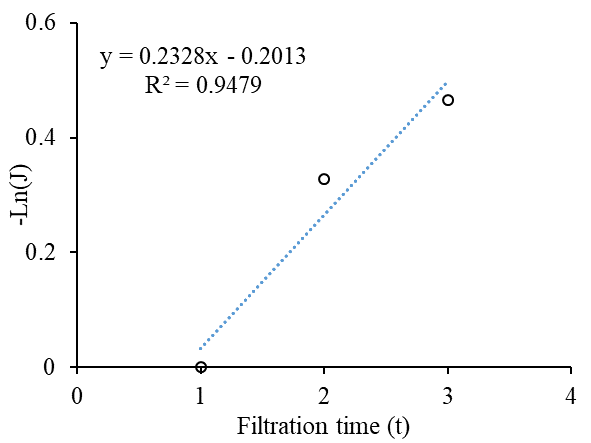

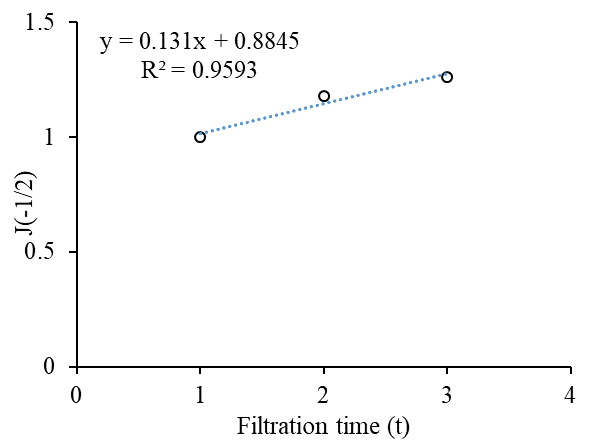

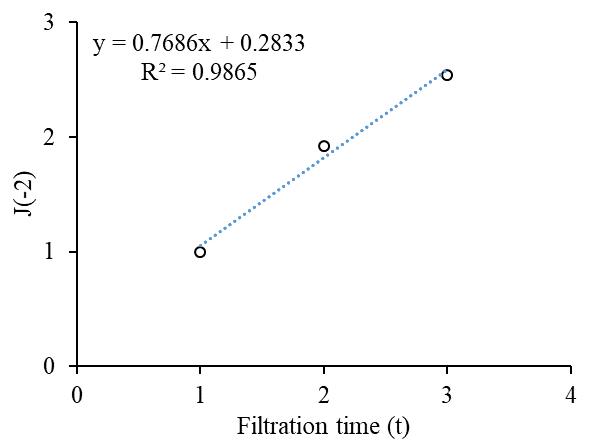


d


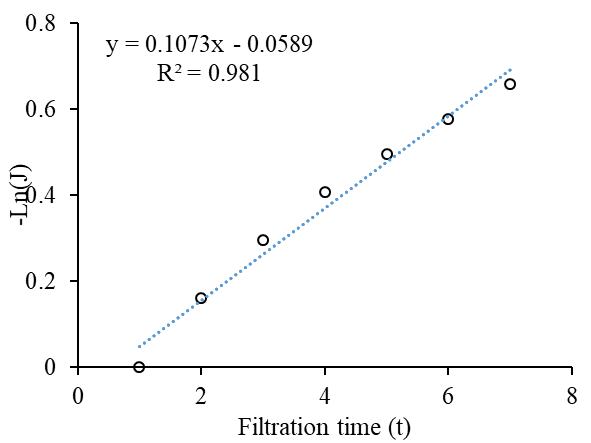

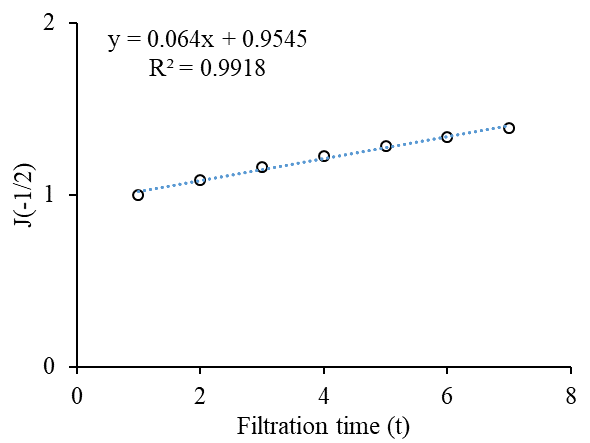

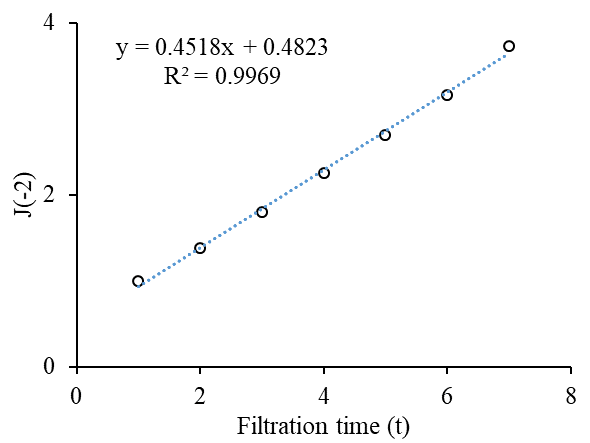


Fig. S3 Linear regression equations according to three fouling models of various MA-IOM fractions, a HPO, b TPI, c C-HPI, d N-HPI


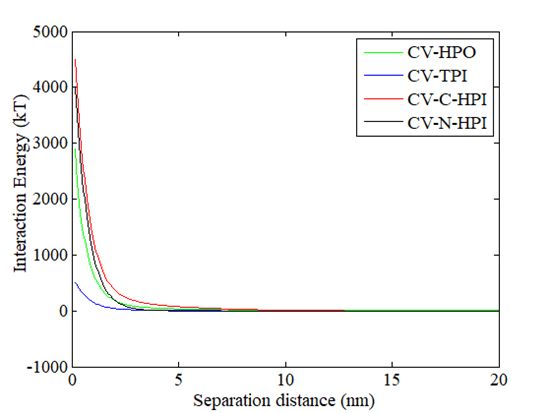


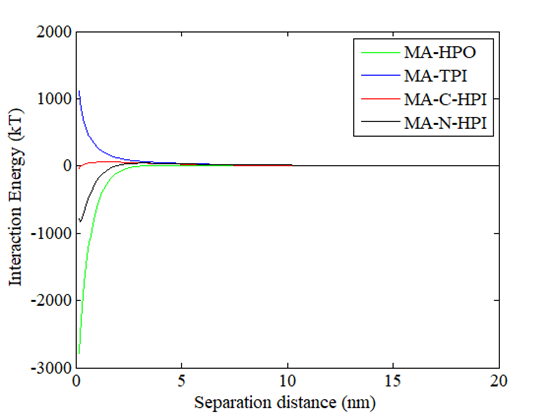


Fig. S4 Interaction energy profiles between the clean membrane surface membrane and CV-IOM and MA-IOM fractions.

Table S1 Surface tension components of clean membrane and foulants

|  | r^LW^ | r^+^ | r^—^ | r^AB^ | r^TOT^ |
| --- | --- | --- | --- | --- | --- |
| clean | 26.1431 | 20.4746 | 21.6536 | 42.1117 | 68.2548 |
| CV-IOM | | | | | |
| HPO | 0.40389 | 18.7021 | 84.9399 | 79.7134 | 80.1173 |
| TPI | 44.3451 | 4.00515 | 97.2743 | 39.4765 | 83.8216 |
| C-HPI | 34.2674 | 29.2216 | 73.7193 | 92.8266 | 127.094 |
| N-HPI | 53.2267 | 35.2688 | 146.985 | 143.999 | 197.2263 |
| MA-IOM | | | | | |
| HPO | 38.1205 | 0.05372 | 15.2426 | 1.80982 | 39.9303 |
| TPI | 3.83913 | 12.2508 | 52.9932 | 50.9592 | 54.7983 |
| C-HPI | 27.7686 | 13.8375 | 34.2405 | 43.5340 | 71.3026 |
| N-HPI | 0.6677 | 16.9867 | 12.1950 | 28.7856 | 29.4532 |
